# The comprehensive changes in soil properties are continuous cropping obstacles associated with American ginseng (*Panax quinquefolius*) cultivation

**DOI:** 10.1038/s41598-021-84436-x

**Published:** 2021-03-03

**Authors:** Chongwei Li, Guozhong Chen, Jianlong Zhang, Ping Zhu, Xinfu Bai, Yuping Hou, Xingxiao Zhang

**Affiliations:** School of Life Sciences, Ludong University, Yantai, 264025 China

**Keywords:** Ecology, Plant sciences

## Abstract

This study aims to verify the time-variant feature of American ginseng (AG) continuous cropping obstacles and to explore the factors impeding continuous cropping. We verified the feature with a plant-soil feedback pot experiment and then investigated the factors by comparing the properties of control soils that had not been previously used for growing ginseng (CS) with those of soils with a 10-year-crop-rotation cycle following the growth of AG (RS). It’s found that the survival rate of AG in RS was lower than that in CS. The RS had lower pH, available potassium content, and urease activity. Additionally, *p*-coumaric, *p*-hydroxybenzoic, vanillic, caffeic, and cinnamic acid levels were lower in RS than in CS, but salicylic acid levels showed the opposite pattern. RS had higher *Rhodanobacter* and lower *Acidothermus*, *Sphingomona*s relative abundances in bacterial community. It’s also found that many bacteria were substantially correlated with phenolic acids and soil physiochemical properties. Results indicate that even after 10-year crop rotation, the negative effects of prior continuous cropping of AG has not been eliminated. The growth of AG can be affected negatively with deterioration of soil physicochemical properties and with lower levels of phenolic acids which promote pathogen reproduction. Probiotics reduction also weighs. Moreover, biotic factors are interrelated with abiotic ones. Therefore, it can be inferred that the comprehensive change of soil properties is the main obstacle for continuous cropping.

## Introduction

Continuous cropping obstacles are a common phenomenon, not only in the cultivation of crops such as apples, melons, eggplants, and peanuts, but are also associated with the cultivation of many medicinal plants such as *Angelica sp.*, *Pinellia sp.*, *Atractylodes sp*.^1^, and *Panax quinquefolius* L. (American ginseng, AG)^2^. AG is a perennial herb that belongs to the family Araliaceae. Clinical research has shown that AG, as a kind of medicinal plant with impressive medicinal properties, has the functions of boosting immune system, sobering up, anti-oxidation, anti-cancer, etc^3–5^. In addition, it’s therapeutic in treatment of SARS, H5N1 and COVID-19, according to the research of traditional Chinese medicine^6,7^. It is often used as prescription drugs in medical treatment and disease prevention. However, serious continuous cropping obstacles restrict the replanting and the yield of AG, resulting in huge economic losses.

Crop rotation is one of the most effective practices to reduce or eliminate continuous cropping obstacles. While continuous cropping obstacles of most plants can be eliminated by employing a favorable rotation strategy^8–10^, those of AG can last for more than 5–10 years or even decades^11,12^. The main detrimental factors to replanting AG in land restored by rotation are not clear.

Many studies have reported that crops under continuous cropping can be adversely affected by allelopathic or autotoxic effects^2,13^. Phenolic acids released during plant growth as the main allelochemicals, are the main continuous cropping obstacles to many terrestrial plants. They have been reported to exert inhibitory effects on many crops and medicinal plants^14,15^. Notably, in a study on the allelopathy of single and mixed phenolic acids on AG^16^, nine phenolic acids including cinnamic, vanillic, and ferulic acids were found to inhibit the growth of AG radicles at concentrations ranging from 0.1 to 10 mmol L^−1^; this inhibition increased with increasing phenolic acid concentration which indicated that phenolic acids had direct inhibitory effects on the growth of AG. However, there are also microorganisms that interact with phenolic acids in soil, especially some AG probiotics (e.g., *Sphingomonas*, *Burkholderia*) and AG pathogens (e.g., *Fusarium oxysporum*, *Phytophthora cactorum*). Kertesz et al. found that phenolic acids can be used as substrates by *Sphingomonas* and *Burkholderia*, enhancing their population growth, and that phenolic acids also play a role in the formation of a stable beneficial microbial community^17^. According to Hu et al.^18^, at a concentration of 100 mg L^−1^, ferulic acid promoted the growth of cucumber fusarium wilt, whereas at 150 mg L^−1^, it showed a significant inhibitory effect. Yuan et al.^19^ found that ferulic acid promoted the growth of *Fusarium oxysporum* at low concentrations, but inhibited its growth at high concentrations. On one hand, phenolic acids can be used as substrates to support beneficial microorganisms, but on the other hand, high phenolic acid concentrations can inhibit the growth of pathogenic bacteria, thereby promoting AG growth. Therefore, higher concentrations of phenolic acids can not only inhibit the growth of AG as allelochemicals, but also promote AG growth by stimulating beneficial microorganisms and reducing harmful microorganisms. The direct and indirect effects of allelochemicals may have opposite effects on plant growth, which are similar to the allelopathic effects of different plants on invasive *Phytolacca americana*^20,21^.

In addition, some studies have also showed that planting AG leads to progressive imbalances in soil microbial communities^22,23^, and the depletion of available nutrients in the soil or soil acidification^24,25^. However, there is little research examining whether these deleterious effects on the microbial community, soil nutrient availability, and soil acidification can be reversed after a period of recovery (here, 10 years). Therefore, it is important to measure soil microbial diversity, community composition, and soil physical and chemical properties after crop-rotation restoration in order to determine which factors influencing AG replanting persist over the course of 10 years. Since previous studies have mostly focused on fungi, equally important bacteria^26–28^ have been often overlooked; therefore, here we focused on the bacterial communities.

In the present study, soil used for planting AG and then subjected to crop rotation for 10 years was collected and used for replanting AG (second age AG seedlings). The growth of AG seedlings in rotation soil (RS) was observed to explore the time-variant feature of continuous cropping obstacles and was compared to that in soil where no AG had been grown [control soil (CS)] to verify if: (1) the change in soil phenolic acid content has an effect on soil microbial community composition, and thereby an indirect effect on AG growth; (2) the change in bacterial community structure mainly reflects an increase in pathogen abundance or a reduction in beneficial bacterial abundance; and (3) soil physicochemical properties and enzyme activities remain unsuitable for AG growth.

## Results

### Pot experiment of AG planting

**Figure 1:**
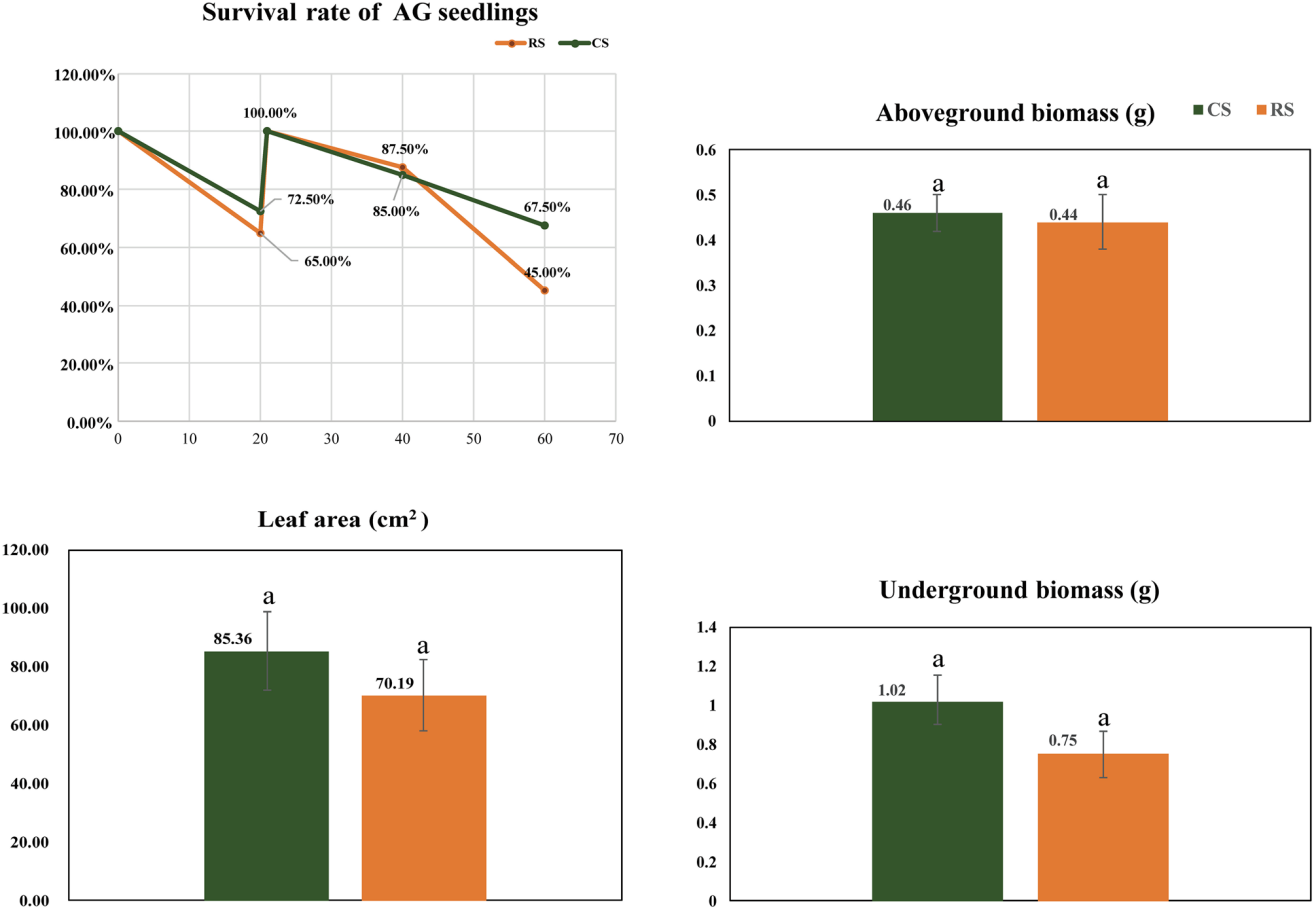
Survival rate and growth index of *Panax quinquefolium* in the pot experiment. The survival rate was calculated every 20 days. The aboveground and underground biomasses were calculated at the end of 60 days. Leaf area was calculated at the final harvest. CS: Plants in the CS group; RS: Plants in the RS group; a: means the difference is not significant (*P* > 0.05). RS: the 10-year post-ginseng rotation soil; CS: soil in which no ginseng was grown before ginseng planting.

As shown in Fig. 1, the survival rate of the two groups decreased in the stable period (20 days)—that of the RS group decreased to 65% and that of the CS group decreased to 72.5%. On the 21st day, the survival rate of the two groups were recovered to 100% by replanting AG seedlings. Although there was no significant difference in leaf area, aboveground biomass, and underground biomass between the two groups, the survival rate of RS was only 45% on the 60th day, while that of CS was 67.5%. The survival rate of AG between the two groups was substantially different.

### Soil physicochemical properties and enzyme activities

The water content of RS was significantly higher than that of CS. While, the pH of RS was significantly lower than that of CS. The content of available K in CS was also significantly (2.96-fold) greater than that in RS. There were no significant differences in the levels of total C, total N, ammonium N, nitrate N, and available P between CS and RS; however, in terms of available nutrients, the levels in CS tended to be higher than those in RS (Table 1).

**Table 1:** Comparison of physicochemical properties of 10-year post-ginseng rotation soil (RS) and soil in which no ginseng was grown (CS) before ginseng planting. Mean ± SE (n = 4) data shown.

Physicochemical properties	RS (ginseng rotation)	CS (no ginseng)
Water content (%)	15.92 ± 0.30*	15.05 ± 0.00
pH	5.00 ± 0.04	5.19 ± 0.04*
Total N (%)	0.21 ± 0.01	0.20 ± 0.02
Total C (%)	1.45 ± 0.45	1.14 ± 0.10
Ammonium N (mg kg^−1^)	12.16 ± 0.56	13.72 ± 0.57
Nitric N (mg kg^−1^)	60.09 ± 3.88	70.50 ± 4.27
Available P (mg kg^−1^)	28.61 ± 6.65	37.83 ± 8.19
Available K (mg kg^−1^)	0.45 ± 0.10	1.33 ± 0.12**

*Statistically significant difference at *P* < 0.05; **P < 0.01 (T test).

There was a significant difference in urease activity between the CS and RS samples, with the activity in CS being 25.13% higher than that in RS; the activities of phosphatase, sucrase, and catalase were higher in CS than in RS, but these differences were not significant (Table 2).

**Table 2:** Comparison of enzyme activities between 10-year post-ginseng rotation soil (RS) and soil in which no ginseng was grown (CS) before ginseng planting.

Soil enzyme activity	RS (ginseng rotation)	CS (no ginseng)
Phosphatase (mg g^−1^)	79.26 ± 34.42	139.96 ± 26.48
Sucrase (mg g^−1^)	0.07 ± 0.01	0.14 ± 0.05
Catalase (mg g^−1^)	224.16 ± 0.19	225.02 ± 0.33
Urease (mg g^−1^)	20.69 ± 0.65	25.89 ± 0.88*

*Statistically significant difference at *P* < 0.05 (T test). Mean ± SE (n = 4) data shown.

### Soil phenolic acids content

**Figure 2:**
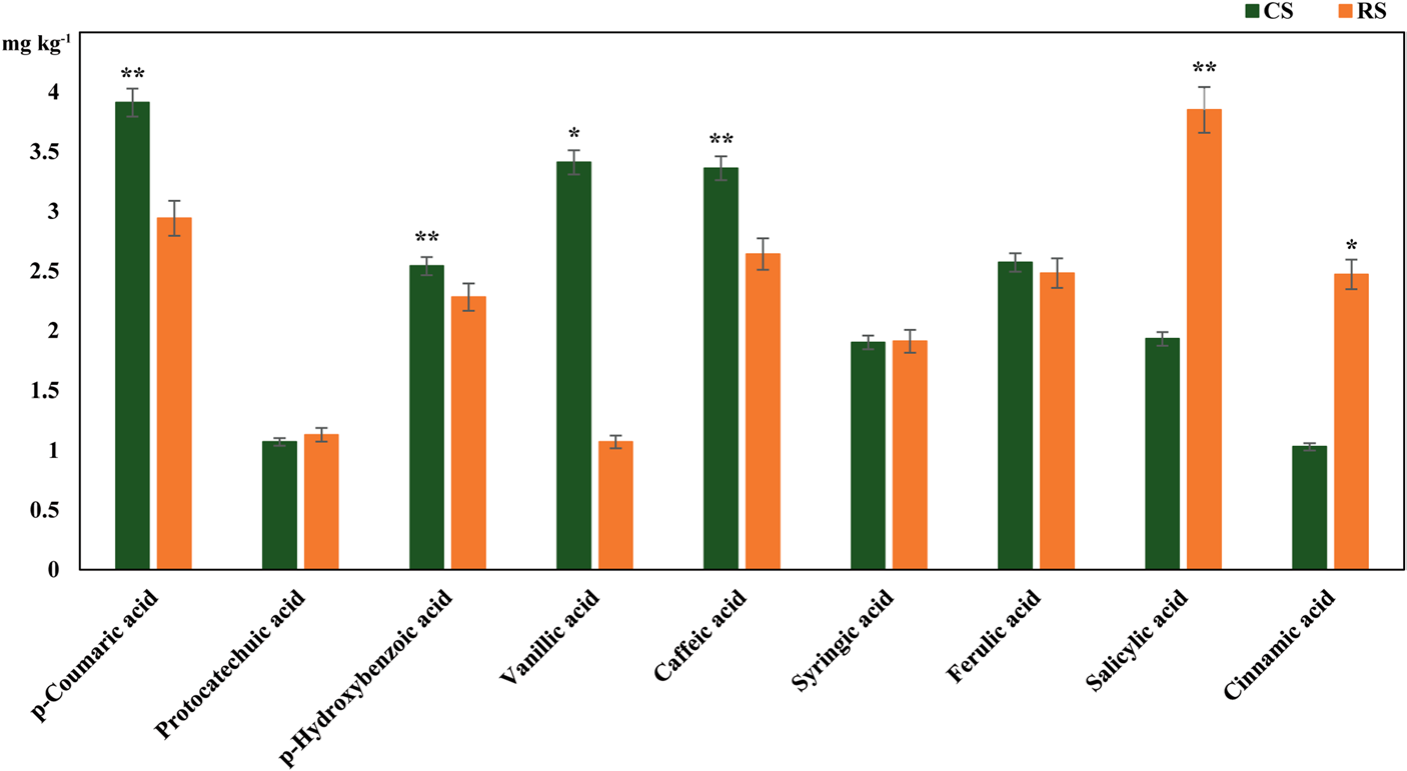
Comparison of phenolic acid content between 10-year post-ginseng rotation soil (RS) and soil in which no ginseng was grown (CS) before ginseng planting. *Represent significant differences (*P* < 0.05) according to Student’s t-test (n = 4). **Represent significant differences (*P* < 0.01).

In terms of phenolic acids, the levels of vanillic and cinnamic acids were markedly higher in CS than those in RS (*P* < 0.05); the levels of *p*-coumaric, caffeic, and *p*-hydroxybenzoic acids were significantly higher in CS than those in RS (*P* < 0.01). However, salicylic acid showed the opposite trend (*P* < 0.01) (Fig. 2).

### Soil bacterial diversity and community structure

#### Sequencing results, OTU cluster analysis, and α-diversity analysis

The V3–V4 region of the 16S rRNA gene was sequenced. After removing the low quality, barcode, and primer sequences, 636,654 effective sequences were obtained from the eight samples; 5421 and 5747 OTUs were contained in RS and CS samples, respectively, and the number of shared OTUs was 3673 after clustering the effective sequences (see Supplementary Figure S5). The slopes of species accumulation curves for bacterial species were flat at different similarity cutoff values, indicating that the identified bacterial diversity was close to saturation and that an increase in the sequencing depth would not increase the number of detected species (see Supplementary Figure S6). Species composition and abundance distributions for each sample, from phylum to genus levels, were obtained using QIIME (see Supplementary Table S2). The diversity and richness of bacterial communities in the soil samples were evaluated and compared using Chao1, Simpson’s, Shannon’s, and ACE indices. Each index reflected higher microbial community diversity and richness in RS than in CS, although none of the differences were significant (see Supplementary Figure S7).

#### β-diversity

**Figure 3:**
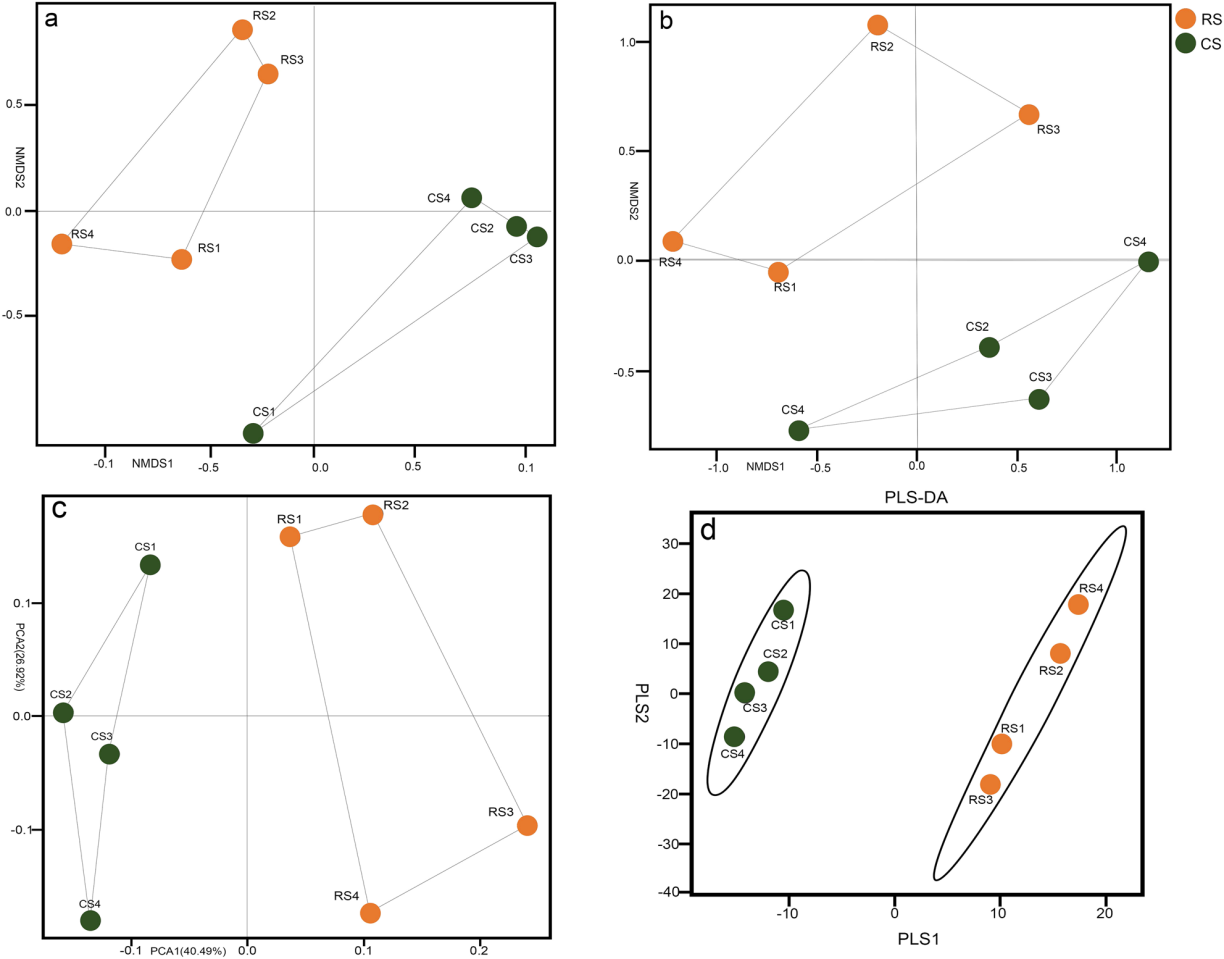
Nonmetric multidimensional scaling (NMDS) ordination plot of bacterial community structure comparing 10-year post-ginseng rotation soil (RS) and soil in which no ginseng was grown (CS) before ginseng planting. (**a**) unweighted and (**b**) weighted UniFrac distance matrix. (**c**) PCA (principal component analysis) of bacterial community structure. (**d**) PLS-DA (partial least squares discriminant analysis) of bacterial community structure. The shorter the distance between two points, the higher the similarity of the microbial community structure between the two groups.

As shown in Table S3, ANOSIM for the unweighted UniFrac result (R = 0.7188, *P* = 0.030) supported the NMDS analysis (Fig. 3a) for the unweighted UniFrac distance matrix, both indicating that the bacterial community structure of RS and CS differed significantly without weighing. However, the weighted UniFrac result (R = 0.3646; *P* = 0.085) indicated that the difference between the two groups of samples was not significant after considering evenness (Fig. 3b). The overall β-diversity index analysis (NMDS, PCA and PLS-DA) showed that there were some differences between RS and CS (Fig. 3a–d).

#### Bacterial community structure and significantly different bacterial composition

**Figure 4:**
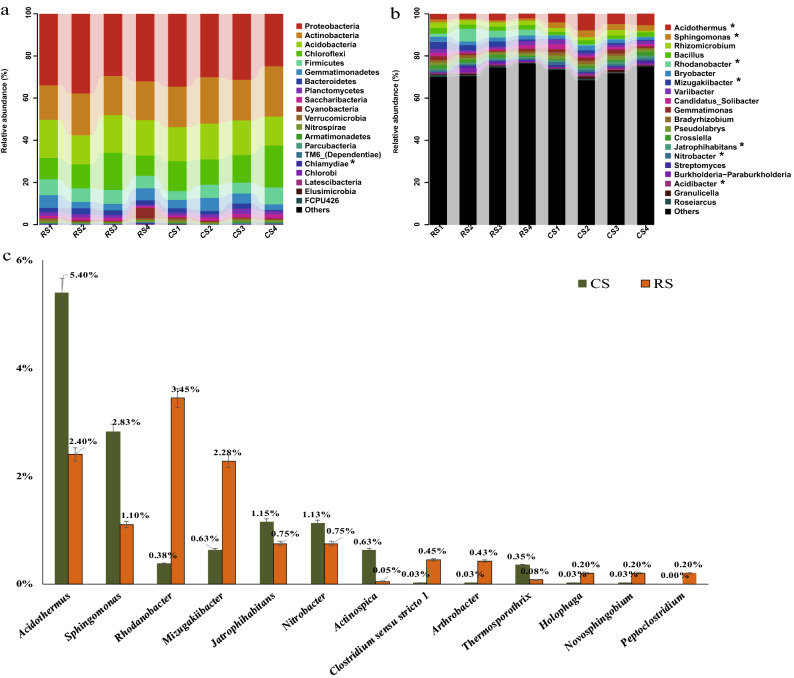
Composition and structure of the bacterial community from the 10-year post-ginseng rotation soil (RS) and soil in which no ginseng was grown (CS) before ginseng planting. (**a**) phylum, (**b**) genus, (**c**) significantly different bacteria at the genus level, comparing RS and CS. The bacteria genera listed in c are all significantly different (t-test, *P* < 0.05). Mean (n = 4) data are shown; error bars represent SE. *represents significant differences (*P* < 0.05) according to the Metastats-test (n = 4).

The bacterial populations in the different samples were analyzed by comparing the obtained 16S rRNA sequences with those of the Greengenes databases using BLAST (Fig. 4). The dominant bacterial phyla were Proteobacteria, Actinobacteria, Acidobacteria, Chloroflexi, Firmicutes, Gemmatimonadetes, and Bacteroidetes across the two groups (Fig. 4a). The most prominent difference between the two groups was that the abundance of Chlamydiae in RS (0.28%) was significantly higher than that in CS (0.10%), and further analysis at lower classification levels revealed that c__Chlamydiae, o__Chlamydiales, f__Simkaniaceae, and g__uncultured were all significantly different (see Supplementary Table S2).

The most abundant bacterial genera were also the same across both groups, namely *Rhizomicrobium*, *Bacillus*, *Bryobacter*, *Variibacter*, *Candidatus_solibacter*, *Gemmatimonas*, *Bradyrhizobium*, *Pseudolabrys*, *Crossiella*, *Streptomyces*, *Burkholderia-Paraburkholderia*, *Granulicella*, and *Roseiarcus* (Fig. 4b). The proportions of *Rhodanobacter* (3.45%), *Mizugakiibacter* (2.28%), *Clostridium*_*sensu*_*stricto*_1 (0.45%), *Arthrobacter* (0.43%), *Holophaga* (0.20%), *Novosphingobium* (0.20%), and *Peptoclostridium* (0.20%) were higher in RS than in CS. The proportions of *Acidothermus* (5.40%), *Sphingomonas* (2.83%), *Jatrophihabitans* (1.15%), *Nitrobacter* (1.13%), *Actinospica* (0.63%), and *Thermosporothrix* (0.35%) were higher in CS than in RS (Fig. 4c).

#### LEfSe analysis of the differentially abundant bacterial communities

**Figure 5:**
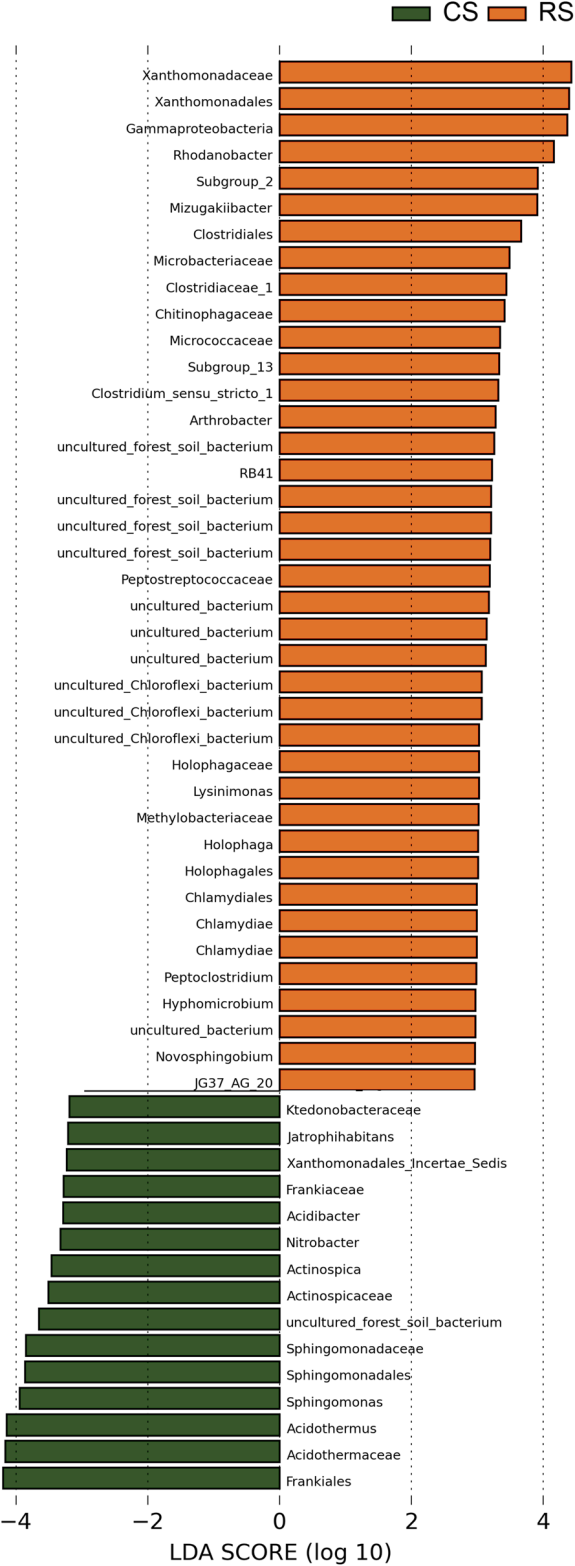
Differentially abundant bacterial taxa as assessed using linear discriminant analysis (LDA) with effect size measurements (LEfSe) in the 10-year post-ginseng rotation soil (RS) and soil in which no ginseng was grown (CS) before ginseng planting.

LEfSe uses LDA scores to estimate the effect size of each differentially abundant taxon, and to rank the relative differences among taxa that are discriminative with biological consistency and statistical significance. The LEfSe analysis of RS and CS bacterial communities showed 42 differentially abundant taxonomic clades with an LDA score higher than 3.0 (Fig. 5). After combining these results (Fig. 5) with the information in Supplementary Table S2, it was found that c (class)_Gammaproteobacteria_o (order)_Xanthomonadales_f (family)_Xanthomonadaceae_g (genus)_*Mizugakiibacter* and g_*Rhodanobacter* (underscores represent relationships at the level of phylogeny); o_Holophagales_f_ Holophagaceae_g_*Holophaga* were all significantly different (T-test, *P* < 0.05) and had a higher abundance in RS. O_Sphingomonadales_f_ Sphingomonadaceae_g_*Sphingomonas* and _g_ *Novosphingobium* were significantly different (t-test, *P* < 0.05); in addition, they had a much higher abundance in CS, except for Novosphingobium. Furthermore, f_Acidothermaceae_g_*Acidothermus* and f_Actinospicaceae_g_*Actinospica* were also significantly different.

#### Correlation analysis

**Figure 6:**
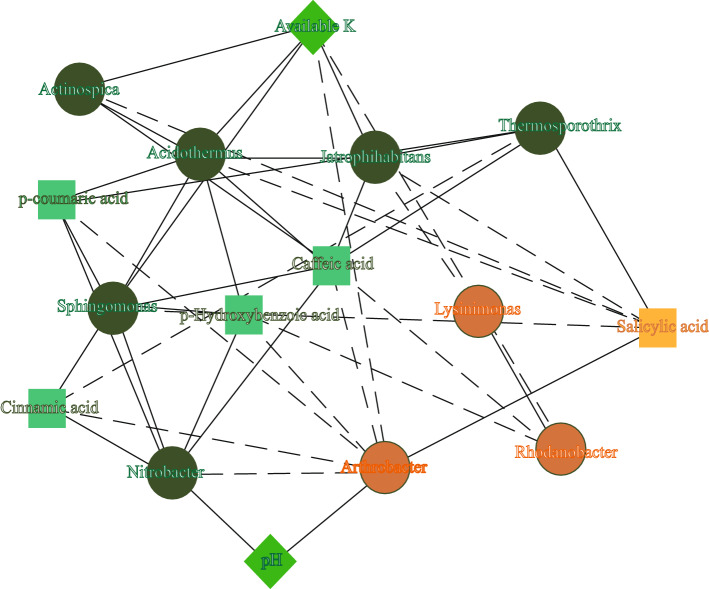
The correlation network of phenolic acids with bacteria and soil physicochemical properties. When the correlation factor is yellow, it means that the factor is enriched in the 10-year post-ginseng rotation soil (RS), whereas when it is green, it represents the soil in which no ginseng was grown (CS) before ginseng planting. The solid line represents the positive correlation among the correlation factors, while the dotted line is the negative correlation. The circle represents bacteria, and the different arrangement of squares represents phenolic acid and physicochemical properties.

To further examine the possible "collaborative" or "competing" relationships among the different communities, Spearman’s rank correlation coefficients between the most abundant genera were calculated using Mothur software. The correlations among the 50 dominant bacterial genera were analyzed (see Supplementary Figure S8), and the correlation analysis between the top ten bacterial genera (see Supplementary Table S4) and factors of interest—comprising six different phenolic acids and three physicochemical properties—indicated a number of interesting relationships. As shown in the Fig. 6 and Supplementary Table S4, *Sphingomonas* with low relative abundance in RS significantly positively correlated with available K, concentration of phenolic acid, *Acidothermus* and *Nitrobacter* abundance and negatively correlated with salicylic acid levels.

## Discussion

### Pot experiment of AG planting

As shown in Fig. 1, compared to CS, the survival rate of 10-year rotation AG decreased, indicating that 2-year-old AG survival rate in RS was lower than that of AG in CS. This confirmed the continued existence of AG continuous cropping obstacles in RS.

### The decrease of physicochemical properties and enzyme activity

Plant growth requires water and nutrients. Because soil physicochemical properties influence water and nutrient availability, changes in soil physicochemical properties directly affect AG growth. In the present study, the water content of RS was significantly higher than that of CS under the same management conditions (Table 1). Shu et al.^29^ found that high soil water content induced root rot disease in AG when sandy loam water content exceeded 30% or that of clay exceeded 50%. Similarly, according to Wang et al.^30^, the incidence of rust rot positively correlated with soil moisture and rainfall. Therefore, high soil water content, caused by changes in soil physicochemical properties, may negatively affect AG replanting. Furthermore, the pH of RS was significantly lower than that of CS (Table 1). According to Rahman and Punja^24^, root rot severity at soil pH 5.05 was greater than that at pH 7.0, indicating that acidic conditions can negatively affect AG health. In addition, the available K content in RS was lower than that in CS (Table 1). Sun^31^ found that AG should be fertilized (N, P, K fertilizer) from emergence to early flowering, when its demand for potassium fertilizer is the highest, suggesting that AG has a high potassium requirement. The levels of ammonium N, nitrate N, available P, and available K, but not of total N and total C, were generally lower in RS than in CS (Table 1), indicating that the cultivation of AG may have long-term negative effects on these soil nutrients. The same trend was observed for soil enzyme activity. Urease, a nickel-containing enzyme, catalyzes the hydrolysis of urea into carbonate and ammonia. Here, urease activity was significantly higher in CS than in RS. Average phosphatase and sucrase activities were also higher in CS than those in RS, although these differences were not significant (Table 2). Yang^32^ also found that the activities of sucrase, urease, and phosphatase decreased during AG cultivation. In summary, compared to that of CS, RS had lower fertility, but higher soil water content and lower pH, two conditions which are conducive to AG disease, and that may, therefore, present obstacles to AG replanting.

### The dual effects of phenolic acids

The results showed that the content of salicylic acid in RS was significantly higher than that in CS. Yang^16^ found that among the various phenolic acids tested, salicylic acid had the strongest inhibitory effect on AG radicle growth. In our study, higher salicylic acid content in RS may have posed direct autotoxicity to AG. As a major defense hormone, salicylic acid has the function of enhancing immune signals and reprogramming defense transcriptomes^33^. After planting AG, the soil salicylic acid content increased, which indicated that AG might release more salicylic acid in the growth process to improve immune response to the surrounding environment. Therefore, the role of salicylic acid in the continuous cropping obstacles to AG cultivation deserves further study.

In addition, we found that the content of most phenolic acids, such as *p*-coumaric, *p*-hydroxybenzoic, vanillic, caffeic, and cinnamic acid, decreased after AG cultivation, and had not returned to the levels in CS even after 10 years of subsequent crop rotation. AG requires a suitable environment for growth. Before germination in spring, the ginseng farmers’ association uses wheat straw to cover the soil, which not only maintains soil temperature and retains soil moisture, but also improves soil quality and promotes the growth of AG seedlings. Jia et al.^34^ detected the increase in ferulic, vanillic, cinnamic, and *p*-hydroxybenzoic acid in a wheat-corn rotation area. In addition, Zheng et al.^35^ found that straw return, a common method for soil improvement, also increased the concentration of phenolic acids in soil. In our study, the increased phenolic acid content in CS relative to RS may have been beneficial to the growth of AG. Similar to our research results, Jiao et al.^36^ also found that the content of phenolic acid substances such as syringic, vanillic, *p*-coumaric, and ferulic acid decreased by 49.1–81% after adding AG root residues (simulating the seasonal AG leaf and fibrous root senescence). Therefore, decreases in the soil contents of some phenolic acids after planting AG may underlie the decline of other soil properties, which is not conducive to the subsequent growth of AG.

As described above, some phenolic acids may be beneficial to the growth of AG; if so, by what mechanism do these beneficial phenolic acids exert their role? Phenolic acids are produced by plants under external stress^37–40^. They do have many beneficial functions, such as antibacterial, antioxidant and so on, which can alleviate the stress of plants^41^. However, with the increase of phenolic acid secretion, some phenolic acids will penetrate into the soil and affect the soil microorganisms. Li et al.^42^ found that cinnamic acid inhibits *Cylindrocarpon destructans* (a pathogen of ginseng) growth at high concentrations, while promoting it at low concentrations. Yang et al.^43^ found that vanillic acid promoted the growth of the pathogens *Rhizoctonia solani* and *Fusarium solani* at low concentrations, but inhibited it at high concentrations; many phenolic acid compounds can inhibit the proliferation of *Phytophthora cactorum* (a pathogenic bacterium that causes AG phytophthora disease) at high concentrations. In addition, Yuan et al.^44^ found that *p*-coumaric acid strongly suppressed the in vitro growth of fungi, significantly reducing the decay caused by *Alternaria alternata*. Therefore, it can be seen that phenolic acids have inhibitory effects on pathogens at higher concentrations. With a decrease in soil phenolic acid content, this inhibitory effect on pathogenic bacteria will be weakened, resulting in an imbalance in the soil microbial composition that affects AG growth performance. Overall, soil phenolic acid content may indirectly affect AG growth performance by affecting soil microorganisms.

### The change in the relative abundance of key bacteria

Our results showed that there was no significant difference in bacterial α-diversity between 10-year post-ginseng RS and CS, but there were differences in β-diversity, which reflects community composition and structure, between CS and RS. In other words, there were significant differences in the relative abundance of key bacteria in the bacterial community, such as Chlamydiae (phylum level, RS: 0.28%, CS: 0.10%, *P* = 0.035), within this phylum, the c_Chlamydiae, o_Chlamydiales, f_Simkaniaceae, and g_uncultured; *Acidothermus* (genus level, RS: 2.40%, CS: 5.40%, *P* = 0.030); Sphingomonadales (order level, CS: 2.98%, RS: 1.68%, *P* = 0.002), Sphingomonadaceae (family level, CS: 2.88%, RS: 1.48%, *P* = 0.004), genera *Novosphingobium* (CS: 0.03%, RS: 0.20%, *P* = 0.035) and *Sphingomonas* (CS: 2.83%, RS: 1.10%, *P* = 0.000); *Rhodanobacter* (CS: 0.38%, RS: 3.45%, *P* = 0.050); *Arthrobacter* (CS: 0.03%, RS: 0.43%, *P* = 0.001); *Mizugakiibacter* (CS: 0.63%, RS: 2.28%, *P* = 0.048); *Jatrophihabitans* (CS: 1.15%, RS: 0.75%, *P* = 0.048); *Pseudomonas* (RS: 0.15%, CS: 0.03%, *P* = 0.029) among others (Fig. 4, see Supplementary Table S2).

There was no difference in soil bacterial α-diversity between RS and CS, which may be due to the recovery of soil bacterial diversity after 10 years of rotation. However, the results of the pot experiment showed that RS still presented continuous cropping obstacles, which indicated that restoring soil microbial α-diversity does not alleviate continuous cropping obstacles for AG. Instead, differences in microbial community composition (i.e., β-diversity), particularly the abundances of bacterial taxa that play key roles, may explain the persistence of AG continuous cropping obstacles in RS after 10 years.

Among the differences in microbial community composition, CS had higher relative abundances of some bacterial genera that may be beneficial bacteria. The genus *Acidothermus* had the highest abundance, and it contained a single species, *A. cellulolyticus*, which is thermophilic, acidophilic, and has the ability to produce many thermostable cellulose-degrading enzymes^45^. Therefore, higher cellulose-degrading capacity might exist in CS than that in RS. *Sphingomonas*, a bacterium with the ability to decompose mono- and polycyclic aromatic compounds, as well as heterocyclic compounds, was more abundant in CS than RS, suggesting that bacterial decay of recalcitrant plant compounds was also higher in CS than RS. In addition, *Sphingomonas* not only decomposes monoaromatic phenolic acids but also improves plant stress resistance, and it is considered a plant probiotic^46^. Similar to our results, Li and Jiang^23^ found that *Jatrophihabitans* relative abundance in soil used for AG for 4 years was significantly (*P* < 0.05) lower than that in soil used for other crops over the same period; therefore, we speculate that AG planting has reduced the abundance of *Jatrophihabitans* as a potential beneficial bacterium in soil. All in all, we found that previous AG planting reduced the relative abundance of some functionally-important bacteria, i.e. those with the abilities to decay cellulose and monocyclic/heterocyclic aromatic compounds, as well as the relative abundance of the plant-beneficial microbe *Sphingomonas* even after 10 years of crop rotation. These changes have negative effects on the maintenance of soil microbial community stability and the promotion of AG growth.

Moreover, there are some genera with higher relative abundance in RS that may be harmful pathogens. Many reports have shown that *Chlamydia* and its phylum Chlamydiae are pathogenic to humans and animals, and their host range may be larger^47^; hence, whether *Chlamydia* contributes to the continuous cropping disorder of AG needs further study. Similar to our research results, in a study conducted by Jiang et al.^48^, the abundance rank for *Rhodanobacter* was healthy root group > root rot group > control group; in addition, compared with CS, there was a higher abundance of *Rhodanobacter* in the soil in which Korean ginseng (*Panax ginseng*) was grown^49^. We also found that this genus might be increased by the influence of Panax plants, which warrants further study. Our results showed that *Arthrobacter* was higher in the RS group, and Jiang et al.^48^ also found that the relative abundance of *Arthrobacter* in the root rot group was higher than that in the healthy root group; therefore, we speculate that *Arthrobacter* might be a factor causing root rot of *P. quinquefolius*, leading to a continuous cropping obstacle to AG growth. Our results showed that the abundance of *Pseudomonas sp.* in RS was higher than that in CS (RS: 0.15%, CS: 0.03%, *P* = 0.029, see Supplementary Table S2). Tan et al.^50^ showed that *Pseudomonas sp.* was the main pathogen causing root rot disease in *P. notoginseng*. In addition, Jiang et al.^48^ also found that *Pseudomonas* is abundant in the rhizosphere soils of diseased ginseng roots. Therefore, it is necessary to further study the effects of *Pseudomonas* species on AG growth. To sum up, the relative abundances of a large number of bacteria that are either confirmed or potentially harmful to other plants increased in RS, which may be an important factor leading to the occurrence of continuous cropping obstacles in the 10-year post-ginseng rotation soil.

As shown in Fig. 6, there are many correlations among the three factors. The abundances of *Acidothermus*, *Sphingomonas*, *Jatrophihabitans*, and *Actinospica* were each positively correlated with that of available K, caffeic acid, and cinnamic acid, but negatively correlated with that of salicylic acid. Therefore, the interactions among phenolic acids, microorganisms, and soil nutrients evidenced possible "synergistic" or "antagonistic" effects within the microecosystem. Overall, these complex relationships are the main reason for AG continuous cropping obstacles, but it is still unknown which of these factors plays the primary role. Finally, *Nitrobacter*, *Actinospica*, *Clostridium *sensu stricto* 1*, *Thermosporothrix*, *Holophaga*, and *Peptoclostridium*, also showed significant differences in abundance between RS and CS (Fig. 4), which also should receive more attention.

## Methods

### Study site

Two study sites were selected in the spring of March 2018; these were two adjacent farmlands in Houjia Town, Wendeng District, Weihai, located 45 m above sea level at 122° 13ʹ 17ʺ E, 37° 4ʹ 34ʺ N, and has a continental monsoon climate. The rotation plot had been used for 4-year AG cultivation, following which it was used for 10 years (rotation soil, RS); the control plot was a cropland in which AG had never been planted (control soil, CS). Each plot was approximately 2 ha in size. For the 10 years of crop rotation, crop types and plot management such as fertilization, watering, and weeding were the same in both the plots. When sampled, both were planted with wheat.

### Soil sampling

Each plot was divided into four subplots. Soil samples were collected by the five-point sampling method in each subplot. After removing surface stones, gravel, humus, and plant residues from each sampling point, approximately 100 g of surface soil (0–20 cm depth) was collected. The five soil samples from each subplot were evenly mixed to produce one soil sample of approximately 500 g. Each soil sample was then divided as follows. Approximately 10 g of soil was transferred into cryogenic vials and placed in a liquid nitrogen tank for low-temperature transportation and preservation; after returning to the laboratory, these vials were refrigerated at − 80 °C for use in high-throughput sequencing. Approximately 200 g of soil was immediately stored in a refrigerator at 4 °C to determine soil water, nitrate nitrogen, and ammonium nitrogen content. Approximately 300 g of soil was air-dried, filtered through a 2 mm sieve, and stored at room temperature (18–25 °C) to determine physical and chemical properties, enzyme activity, and phenolic acid content.

### Pot experiment of plant-soil feedback

A sufficient amount of soil was collected from a depth of 0–20 cm in the same eight subplots of the two plots. The soil collected from each plot was divided into 40 pots (pottery basin, 40 cm in diameter and 30 cm in height), with 10 replicates per subplot. The growth rate of 2-year-old AG was the fastest in all cultivated stages, which was most affected by various soil indicators, so it was most suitable for pot experiment. Two seedlings of this age were planted per pot (enough space for 2-year-old AG to grow) i.e., 40 pots × 2 plots × 2 seedlings. The pots were covered with wheat (*Triticum aestivum*) straw after planting. The materials used for artificial shading facilities and to maintain environmental conditions of local planting are presented in Table 3; the growth of AG seedlings was regularly observed and recorded per 20 days after planting. During the first 20 days, not only the soil effects but also transplanting effects were observed; as for AG, transplanting may lead to fibrous root fracture, thereby causing its death. Hence, we chose to reseed the pots on day 21 to recalculate the survival rate, leaf area, biomass, and other indicators.

**Table 3:** Materials used and environmental conditions of artificial shading facilities.

Item	Specific conditions
Materials	Black shading net, steel pipe, and steel wire were used to construct the shade shed for American ginseng planting
Height	2 m
Construction of shading facilities	A single layer of shading network was adopted to construct the shading facilities
Pot position	In the center of the shade
Light transmittance	18–22%
Temperature	Natural temperature without manual intervention, about 15–25℃
Moisture content	Natural precipitation was utilized during most of the planting process, with occasional artificial watering. Soil moisture content was generally 15–16%, maintained by watering when it was less than 10% and draining when it was higher than 18%
Soil property data	As shown in Table 1

American ginseng is geo-authentic, and the climate of Wendeng area is suitable for its growth.

### Soil physicochemical properties and enzyme activities

Soil pH was measured using a Delta 320 electrode pH meter (Mettler Toledo, USA) in a 1:2.5 (w/v) soil water suspension. Water content was measured by oven-drying the fresh soil samples at 105 °C for 24 h. Total nitrogen (N) and carbon (C) contents were determined using a vario EL III elemental analyzer (Elementar Analysensysteme, Germany). Ammonium N content was determined via the potassium chloride extraction–indigo phenol blue colorimetric method; nitrate N content was determined using the phenol disulfonic acid colorimetric method. Available phosphorus (P) content was determined using the sodium bicarbonate extraction–molybdenum-antimony colorimetric method, and the content of available potassium (K) was determined via flame spectrophotometry^51^. Soil urease and acid phosphatase activities were determined via colorimetry. Catalase activity was determined via potassium permanganate titration. Sucrase activity was determined using sodium thiosulfate titration. The determination of soil enzyme activity was based on the method proposed by Guan^52^.

### Phenolic acids in soil

The method proposed by Hartley and Buchan^53^ was used to extract phenolic acids from soil with the following improvements. Air-dried soil (25 g) was added to 25 mL of 1 mol L^−1^ NaOH solution, left overnight, and then agitated for 30 min on a reciprocal shaker. After centrifugal separation of the soil suspension (5 min, 2860 g, 20 ℃), the supernatant was filtered through filter paper. The suspension was then acidified to pH 2.5 with 12 mol L^−1^ hydrochloric acid. After 2 h, humic acid was removed via centrifugation (same as above), and the supernatant was filtered through a 0.22-μm filter to obtain the final extract. This final extract was analyzed using high performance liquid chromatography (HPLC; see details below) on the e2695 HPLC system (Waters, USA) with a diode array detector using Uranus C18 (250 × 4.6 mm, 5 μm) and guard columns (20 × 4.6 mm, 5 μm). The results were converted to dry soil weight.

Standard phenolic acids (see Supplementary Figure S1) were purchased from Sigma (St. Louis, USA). Detection was performed at 280 nm. Different phenolic acids were identified by their retention times compared to those of the purchased standards (see Supplementary Figure S2). The chromatographic data were recorded and processed using an Empower workstation v1.0 (Waters, USA, www.waters.com/waters/education.htm?eid=134647632&locale=101). Standards of nine phenolic acids were prepared in different concentrations. The standard curve equations of the nine phenolic acids were obtained by considering the peak area of the liquid chromatogram as Y and the sample concentration as X. The concentration of each compound in each soil sample was obtained based on the peak areas (see Supplementary Table S1).

The HPLC separations were conducted as follows: the mobile phase comprised aqueous formic acid solution (0.1%, v/v) and acetonitrile; the column temperature was 30 °C; the injection volume was 20 μL and the flow rate was 1.0 mL min − 1. The gradients and timings were 6–10% acetonitrile (0–16 min), 10–22% (16–36 min), 22–50% (36–46 min), 50–100% (46–48 min), and final hold for 5 min. The column was equilibrated for 10 min between injections.

### Genomic DNA extraction, PCR amplification, and high-throughput amplicon sequencing

Total DNA was extracted from soil samples using the PowerSoil DNA Isolation Kit (MoBio, USA), according to the manufacturer’s protocol. The concentration and purity of the obtained DNA samples were then determined using a UV-1200 UV spectrophotometer (Shanghai Mapada, China) and agarose gel electrophoresis. The V3–V4 region of the 16S rRNA gene was amplified using primers 338F (5′-ACTCCTACGGGAGGCAGCA-3′) and 806R (5′-GGACTACHVGGGTWTCTAAT-3′) for bacterial community analysis^54^. PCR amplification was carried out in a total volume of 25 μL containing 5 μL 5 × reaction buffer, 5 μL 5 × GC buffer, 2 μL dNTPs (2.5 mmol), 1 μL 338F primer (10 μmol), 1 μL 806R primer (10 μmol), 2 μL DNA template, 8.75 μL ddH2O, and 0.25 μL Q5 High-Fidelity DNA Polymerase (NEB, USA)^55^. The PCR protocol consisted of an initial denaturation step at 98 °C for 2 min, followed by 28 cycles of denaturation at 98 °C for 15 s, annealing at 55 °C for 30 s, and extension at 72 °C for 30 s; a final extension at 72 °C was performed for 5 min and reaction mixtures were held at 10 °C until further analysis^56^. Three replicates were used per sample, and these were pooled to minimize PCR bias. PCR products were detected via electrophoresis on 2.0% agarose gels, and fragments of about 450 bp were purified with the AxyPrep DNA Gel Extraction Kit (Axygen, USA). PCR amplicons were quantified using the PicoGreen dsDNA Assay Kit (Invitrogen, USA). Finally, paired-end 2 × 300 bp sequencing of bacterial amplicons was carried out on the MiSeq platform (Illumina, USA) at Personal Biotechnology Co., Ltd (Shanghai, China). The detailed experimental procedure and pipeline chart are shown in Supplementary Figures S3 and S4, respectively.

### Sequence analyses

Using the unique barcodes obtained from the quantitative insights into microbial ecology (QIIME, v1.8.0, qiime.org) pipeline^57^, the raw sequences were assembled for each sample after removing the primer sequences and adaptors. Low quality sequences were filtered out using the criteria proposed by^58,59^. Paired-end reads were assembled using FLASH v1.2.7^60^. After chimera detection, the remaining high-quality sequences were clustered into operational taxonomic units (OTUs) at 97% sequence identity by UCLUST (http://drive5.com/usearch/manual/uclust_algo.html)^61^. A representative sequence was selected from each OTU using default parameters. The OTU taxonomic classification was conducted using the basic local alignment search tool (BLAST, https://blast.ncbi.nlm.nih.gov/Blast.cgi) by comparing the representative sequences set against the Greengenes Database^62^ and retrieving the best hit^63^. An OTU table was further generated to record the abundance of each OTU in each sample and its taxonomy. OTUs containing less than 0.001% of total sequences across all samples were discarded.

To minimize the difference in sequencing depth across samples, an averaged, rounded, and rarefied OTU table was generated by averaging 100 evenly resampled OTU subsets below the 90% minimum sequencing depth for further analyses. Sequence data analyses were mainly performed using the QIIME v1.8.0 and R v3.2.0 (https://www.r-project.org/) packages. Chao1, abundance-based coverage estimator (ACE), Shannon’s, and Simpson’s α-diversity indices were calculated at the OTU level using the OTU table in mothur (v1.25.1, https://mothur.org/). OTU-level ranked abundance curves were generated to compare the richness and evenness of OTUs among samples^57^. To explore variation in bacterial community structures across the analyzed soil samples, unweighted and weighted UniFrac distances were also calculated using R v3.2.0. Nonmetric multidimensional scaling (NMDS) analysis, principal component analysis (PCA), and partial least squares discriminant analysis (PLS-DA), were performed on the distance matrices, and coordinates were used to draw 2D graphical outputs. The linear discriminant analysis (LDA) effect size (LEfSe) method was used to detect differentially abundant taxa across groups using the default parameters through the Galaxy online analytics platform (http://huttenhower.sph.harvard.edu/galaxy/)^64^. The difference in sequence size (i.e., relative abundance) between samples (groups) of each taxon at the phylum and genus levels was compared and tested, using mothur v1.25.1 with the statistical algorithm of Metastats (http://metastats.cbcb.umd.edu/)^65^. Mothur v1.25.1 was also used to calculate Spearman’s rank correlation coefficients among the dominant genera (i.e. those with abundance in the top 50) and to construct the correlation network for the dominant genera whose Rho > 0.6 and *P* value < 0.01. This network was then imported into the Cytoscape software v3.8.0 (http://www.cytoscape.org/)^66^. We used analysis of similarities (ANOSIM) to determine the size of the intra- and inter-group differences by ranking the distance between the samples and evaluated the statistical significance of the differences between the original samples using the permutation test, calculated by QIIME v1.8.0^67,68^.

### Statistical analyses

The data concerning survival rate, leaf area, biomass of AG in each sample group, soil physicochemical properties, enzyme activities, content of phenolic acids, α-diversity indices, and the relative abundances of bacterial taxa (phyla and genera) were compared using independent sample t-tests with significance accepted at *P* < 0.05, as performed in SPSS v19.0 (IBM Corp., USA, www.ibm.com/analytics/spss-statistics-software). Pearson’s correlation analysis was used to identify correlations between bacterial genera and each of the phenolic acids and physicochemical properties. R v3.2.0, Adobe Illustrator CS6 (64 bit, adobe.com) and Photoshop CS6 (64 bit, adobe.com) were used to draw analysis charts and output the results. The structural formula of phenolic acid was obtained from indraw (v5.1.0, http://indrawforweb.integle.com/).

### Data accession

Sequencing raw data can be found in the Sequence Read Archive at the National Center for Biotechnology Information under Bioproject ID PRJNA612151 (https://dataview.ncbi.nlm.nih.gov/object/PRJNA612151?reviewer=lou3qt0rvghbih2cbthrqe76l8).

## Conclusions

Compared to that in CS, the AG survival rate in RS was lower in the pot experiment, showing notable signs of continuous cropping obstacles. We found higher water content and acidification, and lower nutrient levels in RS than in CS; these factors were directly or indirectly unfavorable to AG growth. The lower levels of *p*-coumaric, *p*-hydroxybenzoic, vanillic, caffeic, and cinnamic acids might be among the factors in RS that indirectly reduced AG growth by promoting the growth and reproduction of its pathogens; in contrast, higher salicylic acid content might be one of the factors directly impeding AG growth. Analysis of the bacterial community structure and composition showed that Chlamydia had higher abundance in RS than in CS. In addition, at the genus level, *Acidothermus*, which degrades cellulose, and *Sphingomonas*, which decomposes phenolic acids, had lower abundances in RS than in CS. Hence, according to the correlations we observed, we conclude that complex relationships among water content, pH, phenolic acids, and bacteria play important roles in the continuous cropping obstacles found in RS.

## Supplementary Information

Supplementary Information

## References

[1] Zhang XL, Pan ZG, Zhou XF, Ni WT (2007). Autotoxicity and continuous cropping obstacles: A review. Chin. J. Soil Sci..

[2] He CN (2009). Identification of autotoxic compounds from fibrous roots of *Panax quinquefolium* L. Plant Soil.

[3] Jia L, Zhao YQ, Liang XJ (2009). Current evaluation of the millennium phytomedicine- ginseng (II): Collected chemical entities, modern pharmacology, and clinical applications emanated from traditional Chinese medicine. Curr. Med. Chem..

[4] Qi LW, Wang CZ, Yuan CS (2011). Ginsenosides from American ginseng: Chemical and pharmacological diversity. Phytochemistry.

[5] Szczuka D (2019). American ginseng (*Panax quinquefolium* L.) as a source of bioactive phytochemicals with pro-health properties. Nutrients.

[6] Xue L, Shengming S, Yuqing Z (2020). Research progress of ginseng prescription, ginseng and ginsenoside in prevention and treatment of viral diseases. Chin. Tradit. Herb. Drug.

[7] Yaqian B, Jing M, Yue R, Yanling Z, Yanjiang Q (2020). Discovery of intervention effect of Chinese herbal formulas on COVID-19 pulmonary fibrosis treated by VEGFR and FGFR inhibitors. China J. Chin. Mater. Med..

[8] Acosta-Martinez V, Mikha MM, Vigil MF (2007). Microbial communities and enzyme activities in soils under alternative crop rotations compared to wheat-fallow for the Central Great Plains. Appl. Soil Ecol..

[9] Lupwayi NZ (2007). Soil microbial biomass, functional diversity and enzyme activity in glyphosate-resistant wheat-canola rotations under low-disturbance direct seeding and conventional tillage. Soil Biol. Biochem..

[10] Marcinkeviciene A, Pupaliene R (2009). The influence of crop rotation, catch crop and manure on soil enzyme activities in organic farming. Zemdirbyste.

[11] Bobev SG, Baeyen S, Crepel C, Maes M (2003). First report of phytophthora cactorum on American Ginseng (*Panax quinquefolius*) in Bulgaria. Plant Dis..

[12] Lei F, Zhang A, Zhang Q, Zhang L (2010). Advances in research on allelopathy of ginseng and American ginseng. China J. Chin. Mater. Med,.

[13] Zhang FS, Cao YP (1992). Rhizosphere dynamic processes and plant nutrition. Acta Pedol. Sin..

[14] Zhen WC, Wang XY, Kong JY, Cao KQ (2004). Phenolic acids and their allelopathy in root exudates and saplings of Strawberry. J. Hebei Agric. Univ..

[15] Kong CH, Li HB, Hu F, Xu XH, Wang P (2006). Allelochemicals released by rice roots and residues in soil. Plant Soil.

[16] Yang, J. X. *Allelopathy and Influencing Factors of Phenolic Acids from Panax Quinquefolium* MA.Sc. thesis, China Union Medical University (2009).

[17] Kertesz, M. A., Kawasaki, A. & Stolz, A. in *Taxonomy, Genomics and Ecophysiology of Hydrocarbon-Degrading Microbes* Ch. Chapter 9-1, 1-21 (2018).

[18] Hu YS, Wu K, Li CX, Sun FL, Jia XC (2007). Effects of phenolic compounds on the growth of Cucumis sativus seedlings and Fusarium oxysporum hypha. Chin. J. Ecol..

[19] Yuan F, Zhang CL, Shen QR (2004). Effect and mechanism of phenol compounds in alleviating cucumber Fusarium Wilt. Sci. Agric. Sin..

[20] Chen P, Hou Y, Zhuge Y, Wei W, Huang Q (2019). The effects of soils from different forest types on the growth of the invasive plant *Phytolacca americana*. Forests.

[21] Wei W (2020). Mixed evidence for plant-soil feedbacks in forest invasions. Oecologia.

[22] Dong LL (2017). High-throughput sequencing technology reveals that continuous cropping of American ginseng results in changes in the microbial community in arable soil. Chin. Med..

[23] Li L, Jiang JL (2019). Bacterial community analysis of Panax quinquefolium rhizosphere soil by high-throughput sequencing technology. J. Chin. Med. Mater..

[24] Rahman M, Punja ZK (2005). Factors influencing development of root rot on ginseng caused by *Cylindrocarpon destructans*. Phytopathology.

[25] Farh ME, Kim YJ, Kim YJ, Yang DC (2018). *Cylindrocarpon destructans/Ilyonectria* radicicola-species complex: causative agent of ginseng root-rot disease and rusty symptoms. J .Ginseng Res..

[26] Savary S (2019). The global burden of pathogens and pests on major food crops. Nat. Ecol. Evol..

[27] Fisher MC (2012). Emerging fungal threats to animal, plant and ecosystem health. Nature.

[28] Anderson PK (2004). Emerging infectious diseases of plants: pathogen pollution, climate change and agrotechnology drivers. Trends Ecol. Evol..

[29] Shu QL (1998). Species and main inducible factors of root disease of *Panax quinquefolium* in Anhui province. Plant protection.

[30] Wang G (2003). Study on standardized cultivation technology of American ginseng in Changbai Mountain. Res. Info Trat. Chin. Med..

[31] Sun H (2008). Study on the Law of Nutrient Accumulation of American Ginseng.

[32] Yang AH (2017). Changes of Soil Microbial Community, Nutrients and Enzyme Activities During the Cultivation of American Ginseng and Their Relationships.

[33] Zhou JM, Zhang YL (2020). Plant immunity_danger perception and signaling. Cell J..

[34] Jia JM, Zhang YL (2020). Plant immunity_danger perception and signaling. Cell J..

[35] Zheng HH, Hu XJ, Jia JY, Wu E, Xing JJ (2001). Changes in phenolic acid in plough layer and its effects on the growh and yield of summar corn with returning wheat straw. Chin. J. Eco-Agric..

[36] Jiao XL, Du J, Gao WW (2012). Autotoxicity and promoting: Dual effects of root litter on American ginseng growth. Acta Ecol. Sin..

[37] Jiang SJ (2019). The accumulation of phenolic compounds and increased activities of related enzymes contribute to early defense against walnut blight. Physiol. Mol. Plant Pathol..

[38] Ibanez F, Bang WY, Lombardini L, Cisneros-Zevallos L (2019). Solving the controversy of healthier organic fruit: Leaf wounding triggers distant gene expression response of polyphenol biosynthesis in strawberry fruit (Fragaria x ananassa). Sci. Rep.-UK.

[39] Zoufan P, Azad Z, Ghahfarokhie AR, Kolahi M (2020). Modification of oxidative stress through changes in some indicators related to phenolic metabolism in Malva parviflora exposed to cadmium. Ecotox Environ. Safe.

[40] Miotto-Vilanova L (2019). Impact of Paraburkholderia phytofirmans PsJN on Grapevine Phenolic Metabolism. Int. J. Mol. Sci..

[41] Munir N, Hameed AA, Haq R, Naz S (2019). Biochemical changes in cultivars of sweet oranges infected with citrus tristeza virus. Braz. J. Biol..

[42] Li Z-B, Zhou R-J, Xie Y-J, Fu J-F (2016). Allelopathic effects of phenolic compounds of ginseng root rhizosphere on Cylindrocarpon destructans. Chin. J. Appl. Ecol..

[43] Yang JX, Gao WW (2009). Effects of phenolic allelochemicals on the pathogen of *Panax quiquefolium* L. Chin. Agric. Sci. Bull..

[44] Yuan S (2019). Defense responses, induced by p-coumaric acid and methyl p-coumarate, of jujube (*Ziziphus jujuba* Mill) fruit against black spot rot caused by Alternaria alternata. Agric. Food Chem..

[45] Liu M, Huang H, Bao S, Tong Y (2019). Microbial community structure of soils in Bamenwan mangrove wetland. Sci. Rep.-UK.

[46] Chen DM (2010). Diversity of bacterial community in rhizosphere soils under effects of continuously planting burley tobacco. Chin. J. Appl. Ecol..

[47] Horn M (2008). Chlamydiae as Symbionts in Eukaryotes. Annu. Rev. Microbiol..

[48] Jiang JL (2019). Changes in the soil microbial community are associated with the occurrence of *Panax quinquefolius* L. root rot diseases. Plant Soil.

[49] Weon HY (2007). Rhodanobacter ginsengisoli sp. Nov. and Rhodanobacter terrae sp. Nov., isolated from soil cultivated with Korean ginseng. Int. J. Syst. Evol. Microbiol..

[50] Tan Y (2017). Rhizospheric soil and root endogenous fungal diversity and composition in response to continuous Panax notoginseng cropping practices. Microbiol. Res..

[51] Bao SD (2000). Agrochemical Analysis of Soil.

[52] Guan SY (1986). Soil enzymes and their research methods.

[53] Hartley RD, Buchan H (1979). High-performance liquid chromatography of phenolic acids and aldehydes derived from plants or from the decomposition of organic matter in soil. J. Chromatogr. A.

[54] Dennis KL (2013). Adenomatous polyps are driven by microbe-instigated focal inflammation and are controlled by IL-10-producing T cells. Cancer Res..

[55] Claesson MJ (2009). Comparative analysis of pyrosequencing and a phylogenetic microarray for exploring microbial community structures in the human distal intestine. Plos One.

[56] Scholer A, Jacquiod S, Vestergaard G, Schulz S, Schloter M (2017). Analysis of soil microbial communities based on amplicon sequencing of marker genes. Biol. Fert. Soils.

[57] Caporaso JG (2010). QIIME allows analysis of high-throughput community sequencing data. Nat. Methods.

[58] Gill SR (2006). Metagenomic analysis of the human distal gut microbiome. Science.

[59] Chen H, Jiang W (2014). Application of high-throughput sequencing in understanding human oral microbiome related with health and disease. Front. Microbiol..

[60] Magoc T, Salzberg SL (2011). FLASH: fast length adjustment of short reads to improve genome assemblies. Bioinformatics.

[61] Edgar RC (2010). Search and clustering orders of magnitude faster than BLAST. Bioinformatics.

[62] DeSantis TZ (2006). Greengenes, a chimera-checked 16S rRNA gene database and workbench compatible with ARB. Appl. Environ. Microbiol..

[63] Altschul SF (1997). Gapped BLAST and PSI-BLAST: A new generation of protein database search programs. Nucleic Acids Res..

[64] Segata N (2011). Metagenomic biomarker discovery and explanation. Genome Biol..

[65] White JR, Nagarajan N, Pop M (2009). Statistical methods for detecting differentially abundant features in clinical metagenomic samples. PloS Comput Biol.

[66] Shannon P (2003). Cytoscape: a software environment for integrated models of biomolecular interaction networks. Genome Res..

[67] Clarke KR (1993). Nonparametric multivariate analyses of changes in community structure. Aust. J. Ecol..

[68] Warton DI, Wright ST, Wang Y (2012). Distance-based multivariate analyses confound location and dispersion effects. Method Ecol. Evol..

