# Supplementary material for: The comprehensive changes in soil properties are continuous cropping obstacles associated with American ginseng (Panax quinquefolius) cultivation
Source: Sci Rep. 2021 Mar 3;11:5068. doi: 10.1038/s41598-021-84436-x (PMC7930255; doi:10.1038/s41598-021-84436-x)
Supplement: Supplementary file 1 — Supplementary Information [file 41598_2021_84436_MOESM1_ESM.docx]

The comprehensive changes in soil properties are continuous cropping obstacles associated with American ginseng (*Panax quinquefolius*) cultivation

Chongwei Li†, Guozhong Chen†, Jianlong Zhang, Ping Zhu, Xinfu Bai, Yuping Hou*, and Xingxiao Zhang*

School of Life Sciences, Ludong University, Yantai 264025, China

* Corresponding authors : YP H, hou_yuping@163.com; XX Z, [zhangxingxiao@163.com](mailto:zhangxingxiao@163.com)

†Co-authors contributed equally to this work and should be considered co-first authors.

Figure S1 Structural formula of nine standard phenolic acids.


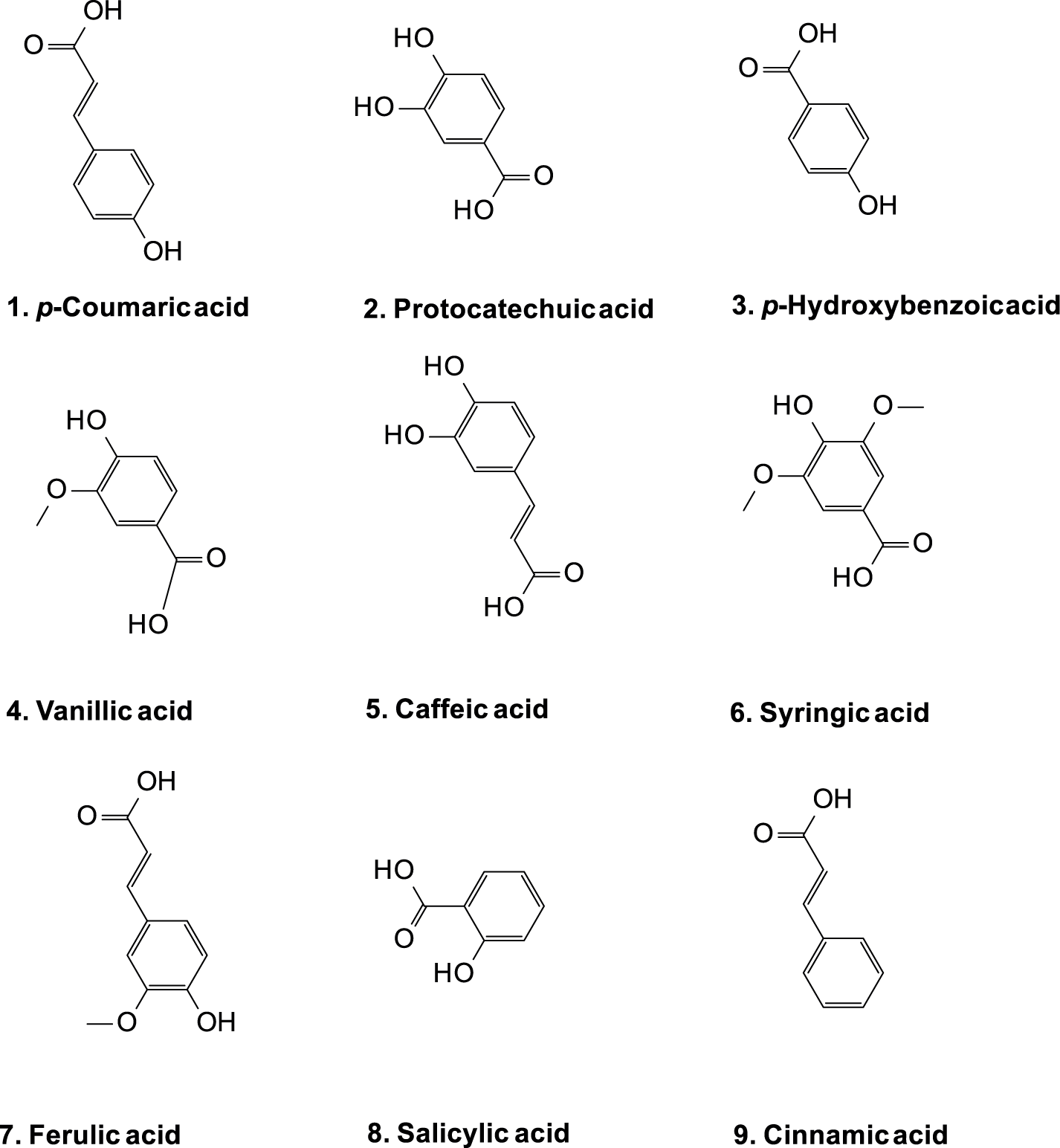


Figure S2 Chromatogram of nine standard phenolic acids at 280 nm. 1: *p*-coumaric acid; 2: protocatechuic acid; 3: *p*-hydroxybenzoic acid; 4: vanillic acid; 5: caffeic acid; 6: syringic acid; 7: ferulic acid; 8: salicylic acid; 9: cinnamic acid. Retetion time of each peak (unit, min): 1–9, 11.16, 12.49, 20.18, 24.90, 26.20, 27.40, 37.51, 43.94, 47.44, respectively.


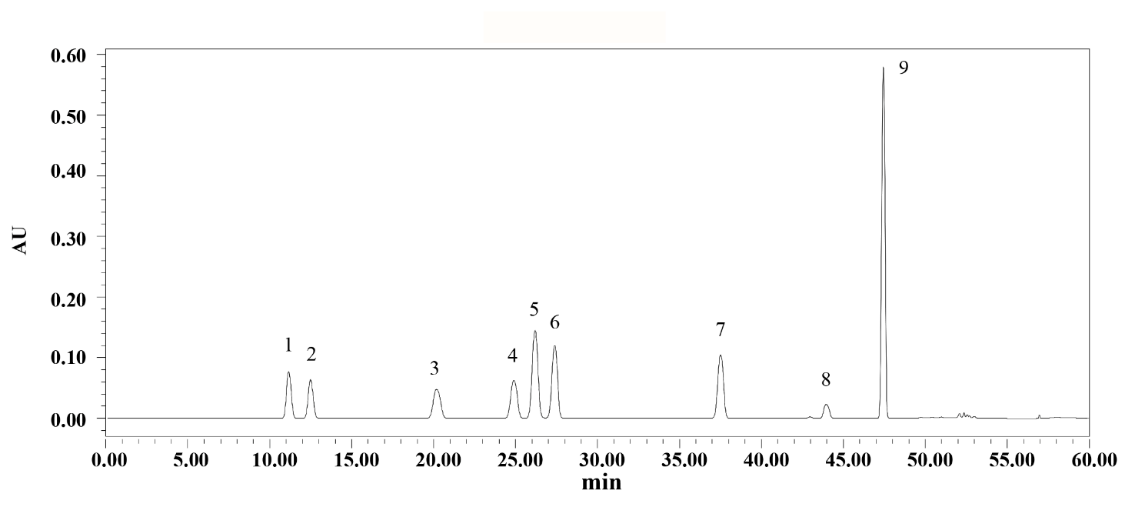


Figure S3 The flow chart of the sequencing experimental procedure.


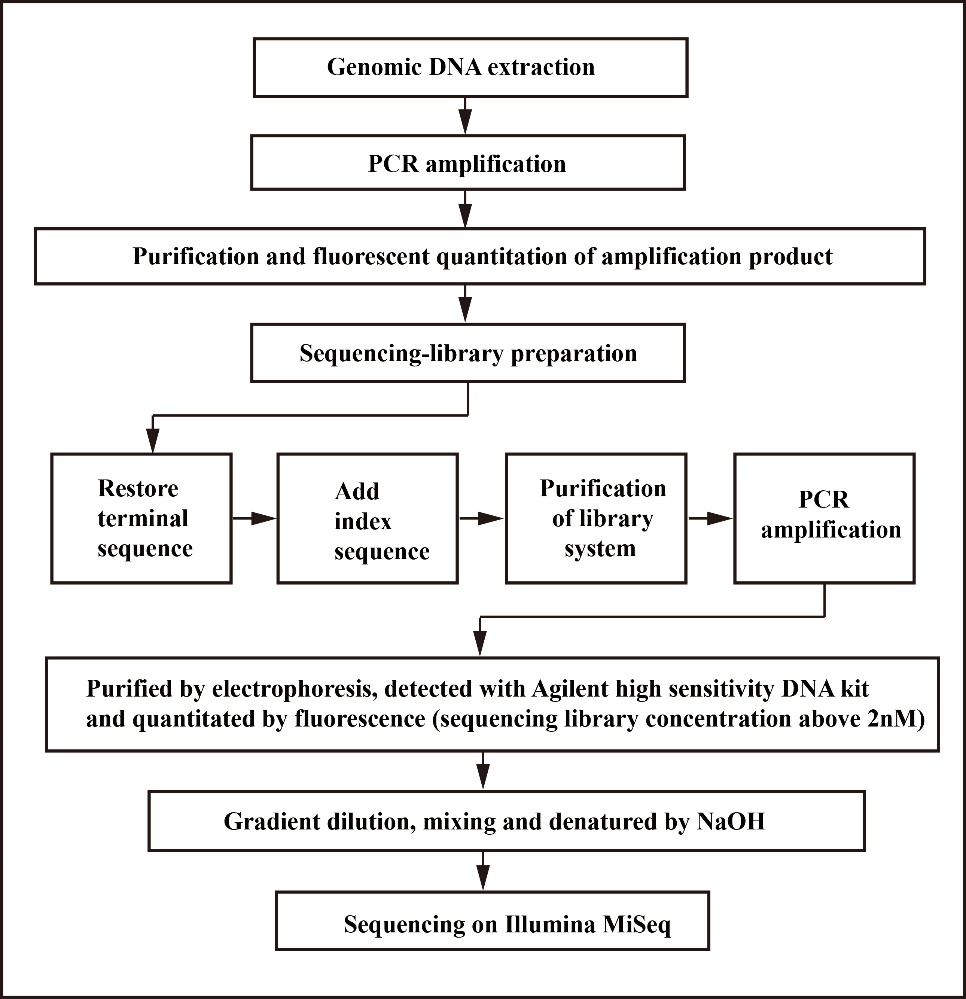


Figure S4 The pipeline chart of the sequences analyses.


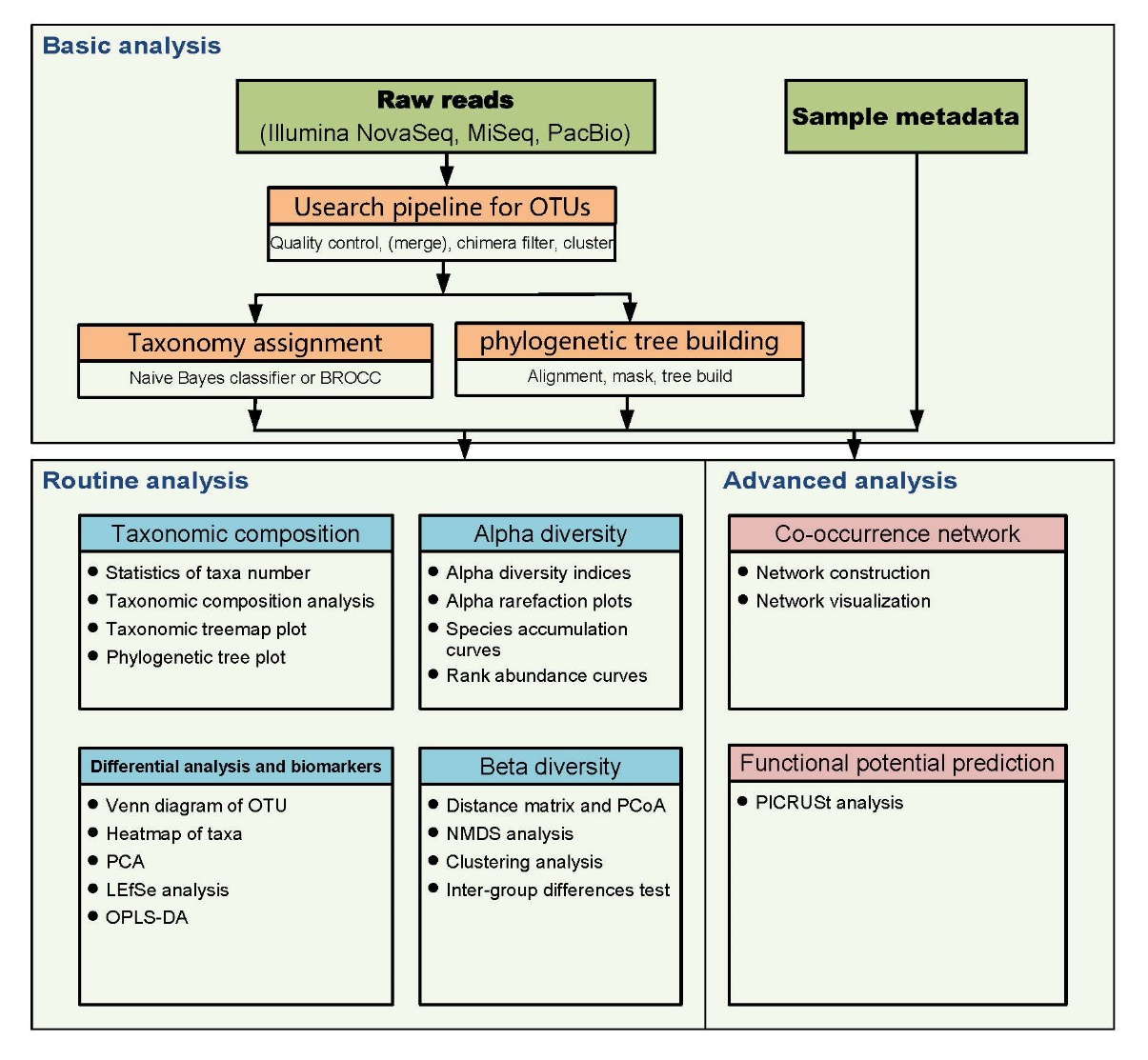


Figure S5 The Venn diagram of OTU. RS: 10-year post-ginseng rotation soil; CS: soil in which no ginseng was grown before ginseng planting. Each ellipse represents one Group samples, the overlapping area between ellipses indicates the shared OTUs among samples groups, and the number of each block indicates the number of shared (or unique) OTUs of the samples groups contained in the block.


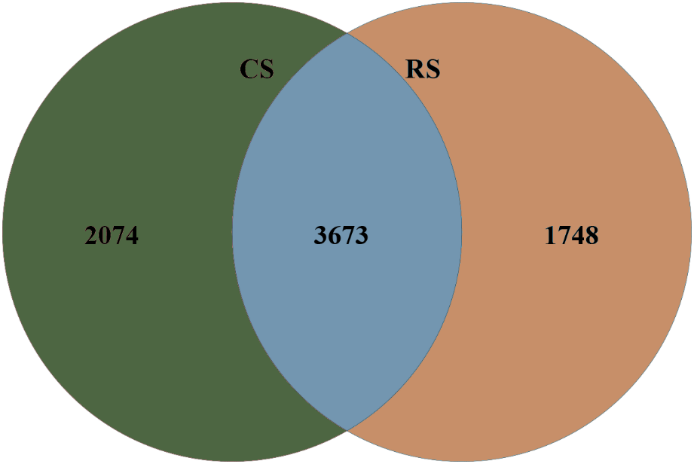


Figure S6 Species accumulation curves measuring the observed species in bacterial communities. The abscissa represents the sample size, the ordinate represents the number of detected objects, and the blue shadow reflects the confidence interval of the curve. The results reflect the increasing rate of new species observed during the process of population sampling. Using the species accumulation curve to judge whether the sample size is large enough, the sharp rise of the curve indicates that the sample size is insufficient and needs to be expanded; otherwise, it indicates that the sample size is enough to reflect the richness of the community. Initially, the number of species increased sharply, and then gradually plateaued, which indicated that the sample size for the sequencing analysis was sufficient.


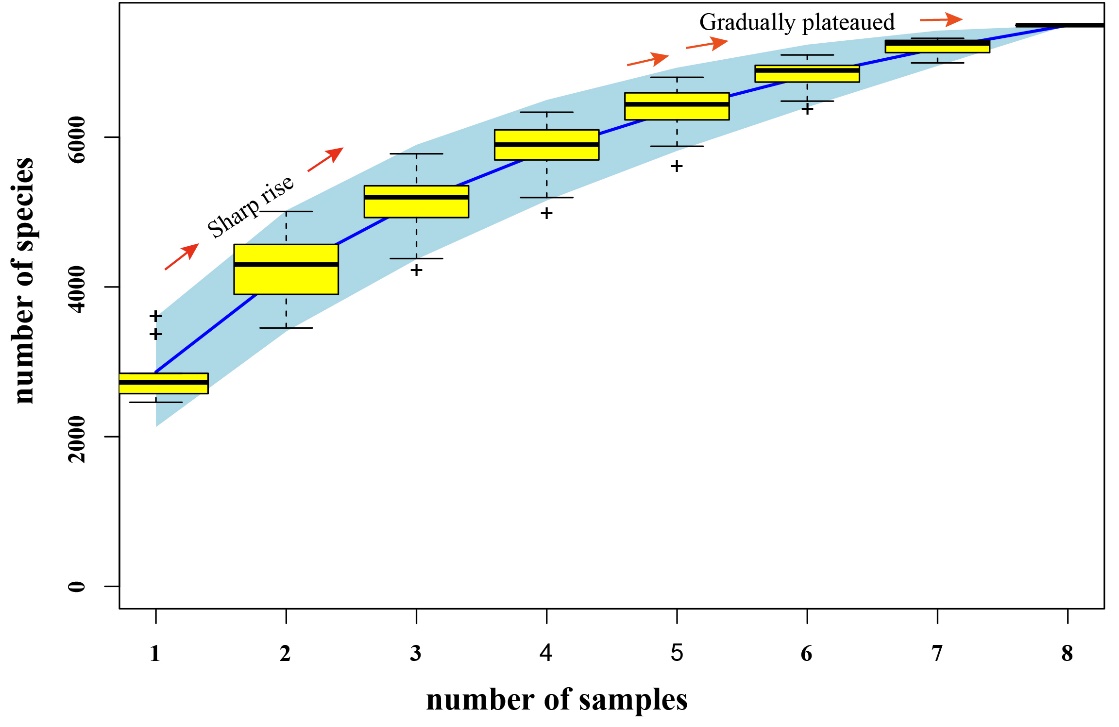


Figure S7 Comparison of four alpha diversity indices (Simpson, Shannon, ACE and Chao1) between 10-year post-ginseng rotation soil (RS) and soil in which no ginseng was grown (CS) before ginseng planting. Mean (n = 4) data shown; error bars represent SE.


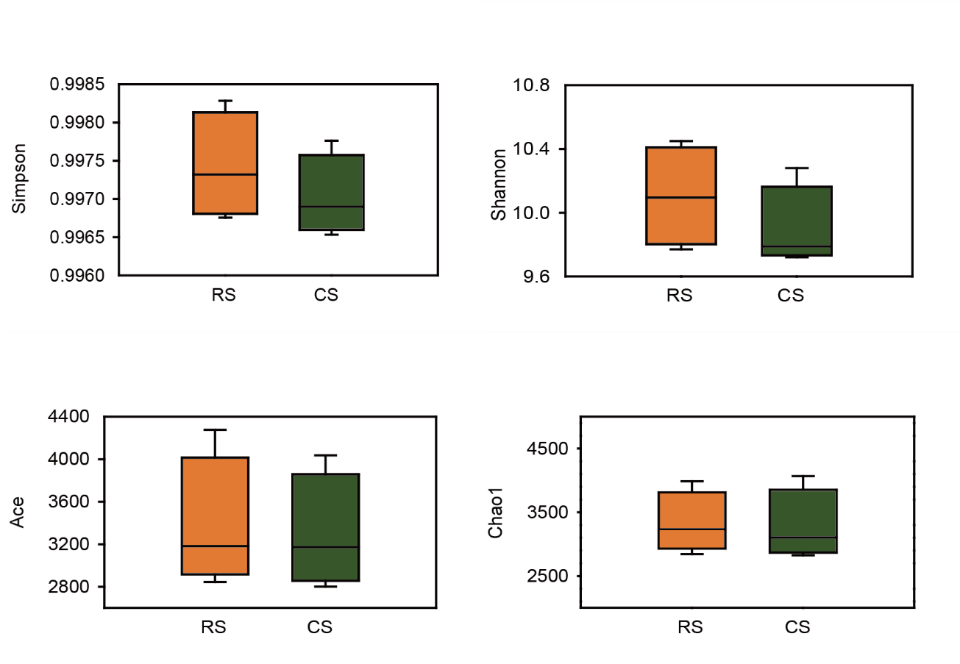


Figure S8 The correlation coefficient of Spearman rank among the top 50 dominant genera was calculated by Spearman correlation network analysis, for which Rho > 0.6 and P value< 0.01 related dominant genera to construct an associated network node represents each dominant genus, identified by different colors, the connection between nodes indicates that there is a correlation between the two genera, the red line indicates a positive correlation, and the green line indicates a negative correlation. The more connections through a node, the more association the genus has with other members of the flora.


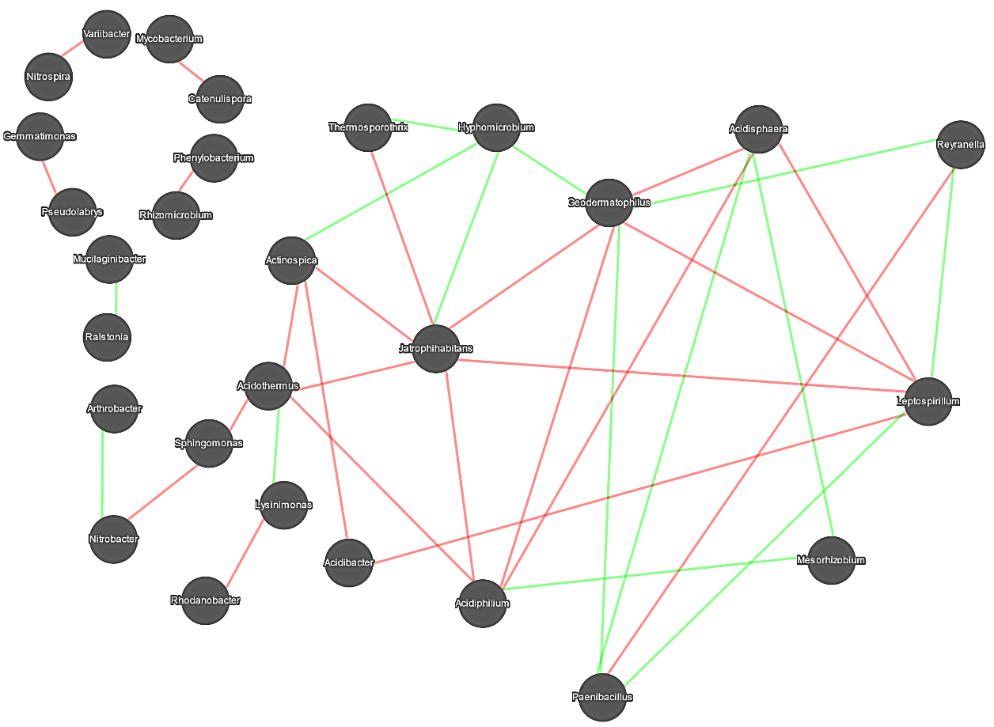


Table S1 Standard curve equations of the nine phenolic compounds.

| Standard substances | Standard curve equations | Regression degree | Linear ranges |
| --- | --- | --- | --- |
| *p*-Coumaric acid | Y = 31530x-28246 | 0.9999 | 0.001–0.1 |
| Protocatechuic acid | Y = 27889x-25769 | 0.9999 | 0.001–0.1 |
| *p*-Hydroxybenzoic acid | Y = 29916x-27528 | 0.9999 | 0.001–0.1 |
| Vanillic acid | Y = 35119x-31949 | 0.9999 | 0.001–0.1 |
| Caffeic acid | Y = 72852x-65440 | 0.9999 | 0.001–0.1 |
| Syringic acid | Y = 57725x-52334 | 0.9999 | 0.001–0.1 |
| Ferulic acid | Y = 52004x-46029 | 0.9999 | 0.001–0.1 |
| Salicylic acid | Y = 10682x-10920 | 0.9999 | 0.001–0.1 |
| Cinnamic acid | Y = 170030x-146718 | 0.9999 | 0.001–0.1 |

Table S2 Comparisons of relative abundance of major lineages in the bacterial communities of 10-year post-ginseng rotation soil (RS) and soil in which no ginseng was grown (CS) before ginseng planting.

| Taxonomy | % relative abundance (mean ± SE) in: | | |  |
| --- | --- | --- | --- | --- |
|  | RS% | CS% | P |  |
| Proteobacteria | 33.23±1.74 | 30.13±2.01 | 0.289 |  |
| Alphaproteobacteria | 18.53 ±1.02 | 19.98± 1.45 | 0.472 |  |
| Sphingomonadales | 1.68±0.00 | 2.98±0.00 | 0.002 |  |
| Sphingomonadaceae | 1.48±0.00 | 2.88±0.00 | 0.004 |  |
| *Novosphingobium* | 0.20±0.00 | 0.03±0.00 | 0.035 |  |
| *Sphingomonas* | 1.10±0.00 | 2.83±0.00 | 0.000 |  |
| Rhizobiales | 10.75±0.01 | 10.03±0.01 | 0.666 |  |
| Bradyrhizobiaceae | 2.03±0.00 | 2.65±0.00 | 0.069 |  |
| Methylobacteriaceae | 0.28±0.00 | 0.08±0.00 | 0.097 |  |
| *uncultured* | 0.13±0.00 | 0.00±0.00 | 0.015 |  |
| *Nitrobacter* | 0.75±0.00 | 1.13±0.00 | 0.038 |  |
| Rhodospirillales | 5.08±0.01 | 6.00± 0.00 | 0.377 |  |
| Caulobacterales | 0.95±0.00 | 0.80±0.00 | 0.432 |  |
| Gammaproteobacteria | 9.08 ± 1.54 | 4.20±0.31 | 0.048 |  |
| Xanthomonadales | 7.83±0.02 | 2.73±0.00 | 0.040 |  |
| Xanthomonadaceae | 6.93±0.01 | 1.33±0.00 | 0.027 |  |
| *Mizugakiibacter* | 2.28±0.00 | 0.63±0.00 | 0.048 |  |
| *Rhodanobacter* | 3.45±1.00 | 0.38±0.06 | 0.050 |  |
| Xanthomonadales_Incertae_Sedis | 0.50±0.00 | 0.83±0.00 | 0.059 |  |
| Legionellales | 0.75±0.00 | 0.68±0.00 | 0.391 |  |
| Enterobacteriales | 0.13±0.00 | 0.50±0.00 | 0.459 |  |
| Pseudomonadales | 0.20±0.00 | 0.13±0.00 | 0.638 |  |
| *Pseudomonas* | 0.15±0.00 | 0.03±0.00 | 0.029 |  |
| HTA4 | 0.08±0.00 | 0.10±0.00 | 0.638 |  |
| Betaproteobacteria | 4.15±0.25 | 4.15±1.26 | 1.000 |  |
| BurkhReriales | 1.68±0.01 | 1.78±0.01 | 0.948 |  |
| Comamonadaceae | 0.35±0.00 | 0.53±0.00 | 0.694 |  |
| *unidentified* | 0.13±0.00 | 0.03±0.00 | 0.030 |  |
| SC-I-84 | 1.78±0.00 | 1.60±0.00 | 0.759 |  |
| Nitrosomonadales | 0.63±0.00 | 0.70±0.00 | 0.841 |  |
| Rhodocyclales | 0.03±0.00 | 0.00±0.00 | 0.391 |  |
| Deltaproteobacteria | 1.48±0.35 | 1.78±0.25 | 0.542 |  |
| Myxococcales | 0.93±0.00 | 1.40±0.00 | 0.237 |  |
| Desulfurellales | 0.43±0.00 | 0.18±0.00 | 0.206 |  |
| Oligoflexales | 0.05±0.00 | 0.08±0.00 | 0.391 |  |
| Bdellovibrionales | 0.08 0.00 | 0.10±0.00 | 0.391 |  |
| Actinobacteria | 18.33±0.72 | 21.08±1.15 | 0.098 |  |
| Actinobacteria | 9.38±1.11 | 12.60±0.66 | 0.049 |  |
| Frankiales | 3.80±0.00 | 7.38±0.01 | 0.023 |  |
| Frankiaceae | 0.78±0.00 | 1.18±0.00 | 0.040 |  |
| *Jatrophihabitans* | 0.75±0.00 | 1.15±0.00 | 0.048 |  |
| Acidothermaceae | 2.40±0.00 | 5.40±0.01 | 0.030 |  |
| *Acidothermus* | 2.40±0.00 | 5.40±0.01 | 0.030 |  |
| Pseudonocardiales | 1.43±0.00 | 1.10±0.00 | 0.528 |  |
| Micrococcales | 1.63±0.00 | 0.85±0.00 | 0.283 |  |
| Micrococcaceae | 0.50±0.00 | 0.08±0.00 | 0.007 |  |
| *Arthrobacter* | 0.43 ±0.00 | 0.03±0.00 | 0.001 |  |
| Streptomycetales | 0.70 ±0.00 | 0.93±0.00 | 0.523 |  |
| Micromonosporales | 0.60± 0.00 | 0.70±0.00 | 0.876 |  |
| Catenulisporales | 0.33±0.00 | 0.95±0.00 | 0.061 |  |
| Actinospicaceae | 0.05±0.00 | 0.63±0.00 | 0.006 |  |
| *Actinospica* | 0.05±0.00 | 0.63±0.00 | 0.049 |  |
| Corynebacteriales | 0.40±0.00 | 0.35±0.00 | 0.769 |  |
| Thermoleophilia | 6.85±0.64 | 6.78±0.68 | 0.906 |  |
| Gaiellales | 5.35±0.01 | 5.23±0.00 | 0.833 |  |
| *uncultured* | 5.20±0.01 | 5.15±0.00 | 0.934 |  |
| Solirubrobacterales | 1.50±0.00 | 1.55±0.00 | 0.752 |  |
| JG30-KF-CM45 | 0.45±0.00 | 0.50±0.00 | 0.752 |  |
| Acidimicrobiia | 1.98±0.09 | 1.63±0.13 | 0.155 |  |
| *Acidimicrobiales* | 1.998±0.00 | 1.63±0.00 | 0.155 |  |
| Acidobacteria | 16.60±1.01 | 15.80±0.72 | 0.544 |  |
| Acidobacteria | 7.28±0.68 | 9.45±0.53 | 0.046 |  |
| Acidobacteriales | 7.28±0.01 | 9.45±0.01 | 0.046 |  |
| *Acidobacteriaceae_(Subgroup_1)* | 7.28±0.00 | 9.45±0.00 | 0.046 |  |
| Solibacteres | 3.13±0.48 | 3.13±0.32 | 1.000 |  |
| Solibacterales | 3.13±0.00 | 3.13±0.00 | 1.000 |  |
| Subgroup_2 | 3.63±0.76 | 1.80±0.01 | 0.081 |  |
| Subgroup_6 | 0.90±0.32 | 1.2±0.14 | 0.881 |  |
| Subgroup_13 | 0.700.11 | 0.25 ± 0.00 | 0.021 |  |
| uncultured_bacterium | 0.45±0.00 | 0.18±0.00 | 0.017 |  |
| *uncultured_bacterium* | 0.45±0.00 | 0.18±0.00 | 0.035 |  |
| Holophagae | 0.40 ± 0.10 | 0.13 ± 0.00 | 0.151 |  |
| Subgroup_7 | 0.18±0.00 | 0.10±0.00 | 0.650 |  |
| Holophagales | 0.23±0.00 | 0.03±0.00 | 0.016 |  |
| Holophagaceae | 0.23±0.00 | 0.03±0.00 | 0.048 |  |
| *Holophaga* | 0.20±0.00 | 0.03±0.00 | 0.048 |  |
| Blastocatellia | 0.48±0.28 | 0.08±0.00 | 0.289 |  |
| Blastocatellales | 0.48±0.00 | 0.08±0.00 | 0.289 |  |
| Chloroflexi | 12.13±0.02 | 14.78±0.02 | 0.333 |  |
| Ktedonobacteria | 7.08±2.04 | 9.53±0.02 | 0.417 |  |
| Ktedonobacterales | 4.78±0.02 | 6.38±0.01 | 0.488 |  |
| Ktedonobacteraceae | 0.18±0.00 | 0.50±0.00 | 0.041 |  |
| *Thermosporotrichaceae* | 0.15±0.00 | 0.65±0.00 | 0.038 |  |
| *Thermosporothrix* | 0.08±0.00 | 0.35±0.00 | 0.074 |  |
| JG30-KF-AS9 | 1.23±0.00 | 1.58±0.00 | 0.271 |  |
| KD4-96 | 1.53±0.38 | 1.40±0.01 | 0.851 |  |
| JG37-AG-4 | 1.00±0.30 | 1.43±0.00 | 0.547 |  |
| TK10 | 0.78±0.12 | 0.60±0.00 | 0.412 |  |
| unidentified | 0.08±0.00 | 0.28±0.00 | 0.016 |  |
| unidentified | 0.08±0.00 | 0.28±0.00 | 0.016 |  |
| JG30-KF-CM66 | 0.60±0.10 | 0.70±0.00 | 0.658 |  |
| Firmicutes | 6.63±0.01 | 5.85±0.02 | 0.443 |  |
| Bacilli | 3.73±0.37 | 2.98±0.00 | 0.179 |  |
| Bacillales | 3.70±0.00 | 2.95±0.00 | 0.206 |  |
| Clostridia | 2.78±0.32 | 2.85±0.01 | 0.927 |  |
| Clostridiales | 1.25±0.00 | 0.35±0.00 | 0.012 |  |
| *Peptostreptococcaceae* | 0.35±0.00 | 0.00±0.00 | 0.027 |  |
| *Peptoclostridium* | 0.20±0.00 | 0.00±0.00 | 0.016 |  |
| Clostridiaceae_1 | 0.75±0.00 | 0.18±0.00 | 0.011 |  |
| *Clostridium_sensu_stricto_1* | 0.45±0.00 | 0.03±0.00 | 0.023 |  |
| Halanaerobiales | 1.45±0.00 | 2.40±0.01 | 0.301 |  |
| Thermoanaerobacterales | 0.05±0.00 | 0.10±0.00 | 0.182 |  |
| Erysipelotrichia | 0.13±0.05 | 0.03±0.00 | 0.252 |  |
| Erysipelotrichales | 0.13±0.00 | 0.03±0.00 | 0.252 |  |
| Gemmatimonadetes | 4.45±0.02 | 4.30±0.01 | 0.929 |  |
| Gemmatimonaetes | 4.45±0.84 | 4.30±0.73 | 0.897 |  |
| Gemmatimonadales | 4.45±0.02 | 4.30±0.01 | 0.897 |  |
| *Gemmatimonadaceae* | 4.45±0.02 | 4.30±0.01 | 0.897 |  |
| Bacteroidetes | 2.05±0.00 | 1.60±0.00 | 0.288 |  |
| Sphingobacteriia | 1.93±0.11 | 1.43±0.00 | 0.247 |  |
| Sphingobacteriales | 1.93±0.00 | 1.43±0.00 | 0.247 |  |
| *Chitinophagaceae* | 1.20±0.00 | 0.65±0.00 | 0.032 |  |
| *uncultured* | 0.98±0.00 | 0.53±0.00 | 0.049 |  |
| Cytophagia | 0.13±0.09 | 0.15±0.00 | 0.761 |  |
| Cytophagales | 0.13±0.00 | 0.15±0.00 | 0.761 |  |
| Flavobacteriia | 0.05±0.05 | 0.05±0.00 | 1.000 |  |
| Planctomycetes | 1.30±0.00 | 1.63±0.00 | 0.301 |  |
| Planctomycetacia | 1.23±0.23 | 1.55±0.00 | 0.437 |  |
| Saccharibacteria | 1.15±0.00 | 1.53±0.00 | 0.270 |  |
| uncultured_bacterium | 1.00±0.15 | 1.18±0.00 | 0.574 |  |
| soil_bacterium_WF55 | 0.00±0.00 | 0.10±0.00 | 0.252 |  |
| Cyanobacteria | 1.50±0.01 | 0.53±0.00 | 0.432 |  |
| Cyanobacteria | 1.00±1.00 | 0.00±0.00 | 0.391 |  |
| Chloroplast | 0.35±0.10 | 0.30±0.00 | 0.718 |  |
| Verrucomicrobia | 0.65±0.00 | 0.63±0.00 | 0.932 |  |
| Spartobacteria | 0.43±0.18 | 0.25±0.00 | 0.188 |  |
| OPB35_soil_group | 0.15±0.03 | 0.30±0.00 | 0.339 |  |
| Opitutae | 0.03±0.03 | 0.08±0.00 | 0.495 |  |
| Nitrospirae | 0.58±0.00 | 0.65 ± 0.00 | 0.215 |  |
| Nitrospira | 0.58±0.10 | 0.65 0.00 | 0.215 |  |
| Nitrospirales | 0.56±0.00 | 0.65±0.00 | 0.215 |  |
| Nitrospiraceae | 0.48±0.00 | 0.65±0.00 | 0.076 |  |
| Armatimonadetes | 0.23±0.00 | 0.48±0.00 | 0.127 |  |
| Chthonomonadetes | 0.13±0.03 | 0.23±0.00 | 0.308 |  |
| Armatimonadia | 0.03±0.03 | 0.10±0.00 | 0.319 |  |
| Chlamydiae | 0.28±0.00 | 0.10±0.00 | 0.035 |  |
| Chlamydiae | 0.28±0.00 | 0.10±0.00 | 0.035 |  |
| Chlamydiales | 0.28±0.00 | 0.10±0.00 | 0.035 |  |
| Simkaniaceae | 0.15±0.00 | 0.03±0.00 | 0.015 |  |
| *uncultured* | 0.15±0.00 | 0.03±0.00 | 0.017 |  |
| Chlorobi | 0.05±0.00 | 0.13±0.00 | 0.215 |  |
| Chlorobia | 0.05±0.00 | 0.13±0.00 | 0.215 |  |

Table S3 UniFrac analysis of similarities between the bacterial communities present in 10-year post-ginseng rotation soil (RS) and soil in which no ginseng was grown (CS) before ginseng planting. The R value is the statistics of Anosim, which is between - 1 and 1, indicating the difference between the differences between groups and within groups. The closer r value is to 1, the greater the difference between groups, and the smaller the difference within groups, the better the grouping effect; if r = 0, it means that the grouping effect of samples is equal to random allocation, and there is no observable statistical difference between sample groups; if R is negative, it means that the difference within groups exceeds the difference between groups, indicating that the grouping effect is poor. P value reflects the statistical significance of anosim analysis results. The smaller the p value is, the higher the significance of the difference between the sample groups is.

| Method name | R statistic | P value | Number of permutations |
| --- | --- | --- | --- |
| Unweighted | 0.7188 | 0.030 | 999 |
| Weighted | 0.3646 | 0.085 | 999 |

Table S4 R values of Pearson correlation analysis between the top 10 most abundant bacterial genera rank order and the phenolic compounds and physicochemical properties of 10-year post-ginseng rotation soil (RS) and soil in which no ginseng was grown (CS) before ginseng planting. * Statistically significant correlation at P < 0.05; ** P < 0.01. Mean ± SE (n = 4) data shown. Missing values represent no significant correlation.

| Bacterial genus | Phenolic Compounds | | | | | | Physicochemical Properties | | | | |
| --- | --- | --- | --- | --- | --- | --- | --- | --- | --- | --- | --- |
|  | *p*-hydroxybenzoic acid | Caffeic acid | Cinna- mic acid | Salicylic acid | *p*-coumaric acid |  | |  | Available K | pH | Water Content |
| *Acidothermus* | 0.751^*^ | 0.796^*^ | - | -0.737^*^ | 0.864^**^ |  | |  | 0.888^**^ | - | - |
| *Sphingomonas* | 0.847^**^ | 0.946^**^ | 0.792^*^ | -0.877^**^ | 0.919^**^ |  | |  | 0.953^**^ | - | - |
| *Rhodanobacter* | -0.771^*^ | -0.752^*^ | - | - | - |  | |  | -0.863^*^ | - | - |
| *Mizugakiibacter* | - | -0.749^*^ | - | - | - |  | |  | - | - | - |
| *Jatrophihabitans* | - | 0.759^*^ | - | -0.800^*^ | - |  | |  | 0.747^*^ | - | -0.773^*^ |
| *Nitrobacter* | 0.946^**^ | 0.758^*^ | 0.865^**^ | - | 0.868^**^ |  | |  | - | 0.790^*^ | - |
| *Actinospica* | - | 0.736^*^ | - | -0.792^*^ | - |  | |  | 0.707^*^ | - | - |
| *Clostridium sensu stricto 1* | -0.736^*^ | -0.772^*^ | -0.853^**^ | - | - |  | |  | -0.817^*^ | - | 0.792^*^ |
| *Thermosporothrix* | - | 0.762^*^ | -0.775^*^ | 0.804^*^ | 0.757^*^ |  | |  | - | - | - |
| *Arthrobacter* | -0.803^*^ | -0.895^**^ | -0.907^**^ | 0.825^*^ | -0.810^*^ |  | |  | -0.858^**^ | 0.825^*^ | - |
